# Investigation of ribociclib, abemaciclib and palbociclib resistance in ER+ breast cancer cells reveal potential therapeutic opportunities

**DOI:** 10.1038/s41598-025-11052-4

**Published:** 2025-08-05

**Authors:** Mashael Algethami, Ahmed Shoqafi, Ayat Lashen, Shatha Alqahtani, Jake Spicer, Ahmad ALtayyar, Çağla Tosun, Jennie N Jeyapalan, Nigel P. Mongan, Victoria James, Emad A. Rakha, Srinivasan Madhusudan

**Affiliations:** 1 Biodiscovery Institute, School of Medicine, University of Nottingham, University Park, Nottingham, NG7 3RD UK; 2 Department of Pathology, Nottingham University Hospital, City Campus, Hucknall Road, Nottingham, NG51PB UK; 3 Faculty of Medicine and Health Sciences, Centre for Cancer Sciences, University of Nottingham, Sutton Bonington Campus, Sutton Bonington, Leicestershire LE12 5RD UK; 4 Department of Pharmacology, Weill Cornell Medicine, New York, NY 10065 USA; 5 Department of Oncology, Nottingham University Hospitals, Nottingham, NG51PB UK

**Keywords:** CDK4, CDK6, Palbociclib, Ribociclib, Abemaciclib, Mechanism of resistance, Breast cancer, Cancer, Chemical biology, Drug discovery

## Abstract

The cyclin-dependent kinase 4/6 inhibitor (CDK4/6i) ribociclib, abemaciclib, and palbociclib have transformed outcomes in patients with ER+ /HER2 − advanced breast cancer (BC). However, most patients eventually progress, and therapeutic options beyond CDK4/6i are an area of ongoing investigation. Here, we generated and evaluated ribociclib, abemaciclib, and palbociclib-resistant BCs. MCF7 and T47D (ER+ /HER2−) cells were chronically treated with increasing doses of ribociclib (R), abemaciclib (A), or palbociclib (P) over 8 months (0-600 nM). CDK4/6i-resistant cell lines (MCF7rR, MCF7rA, MCF7rP, T47DrR, T47DrA, and T47DrP) were isolated and evaluated for their aggressive phenotypes, cross-resistance, transcriptomic changes, and sensitivity to volasertib (PLK1 inhibitor) and barasertib (AukB inhibitor). Immunohistochemical evaluation of CDK4, CDK6, and p53 (*n* = 1005) and transcriptomic evaluation of *AukB* and *PLK1* were performed in 5031 clinical breast cancers. MCF7rR, MCF7rA, MCF7rP, T47DrR, T47DrA, and T47DrP cells manifested aggressive phenotypes such as increased spheroid formation, invasion, proliferation, and progression through the G1/S phase of the cell cycle despite CDK4/6i treatment, increased resistance to apoptosis, and cross-resistance to other CDK4/6i. Transcriptomic analysis revealed the enrichment of distinct pathways in resistant cells, particularly the upregulation of cell cycle regulatory genes such as *PLK1*, *AukB*, *CDKN2B* and *TGFβ*. *PLK1* or *AukB* overexpressing resistant cells were sensitive to volasertib (PLK1 inhibitor) and barasertib (AukB inhibitor) therapy, which was associated with G2/M cell cycle arrest and increased apoptosis. We conclude that cell cycle upregulation leading to G2/M progression is a key route for CDK4/6i resistance. AukB or PLK1 inhibitors that block G2/M phase could be a promising strategy.

**Supplementary Information:**

The online version contains supplementary material available at 10.1038/s41598-025-11052-4.

## Introduction

Cyclin-dependent kinases (CDKs) are serine/threonine kinases critical for cell cycle regulation^1^. CDK4 and CDK6 regulate G1/S phase progression via retinoblastoma (Rb) signaling^2–4^. Upon mitogenic stimulation, the CDK4-cyclin D1 complex translocates to the nucleus and phosphorylates Rb protein. Rb1 phosphorylation leads to de-repression of the E2F transcription factor, which stimulates protein synthesis required for promoting cell cycle progression and cellular proliferation^2–4^. Cyclin D1 overexpression in breast cancer (BC) can result in constitutive activation of the CDK-cyclin pathway^5^. In addition to the nuclear function of CDK4 or CDK6, its cytoplasmic role has also been described recently. Cancer cell migration and invasion may be promoted by cytoplasmic CDK4-cyclin D1^6^. In addition to G1/S cell cycle regulation, CDK6 transcriptionally regulates vascular endothelial growth factor A (VEGFA, critical for tumor angiogenesis)^7^ and FMS-like tyrosine kinase 3 (FLT3), which is involved in cancer cell proliferation)^8^.

Ribociclib, palbociclib, and abemaciclib are highly potent CDK4/6 inhibitors (CDK4/6i), which target the ATP-binding domains of CDK4 and CDK6. Ribociclib, palbociclib, and abemaciclib induce G1 cell cycle arrest in an Rb-dependent manner^9,10^. Although highly potent, ribociclib and abemaciclib preferentially block (in biochemical assays) CDK4 [ribociclib: IC_50_ = 10 nM, abemaciclib IC_50_ = 2 nM] compared to CDK6 [ribociclib: IC_50_ = 39 nM, abemaciclib IC_50_ = 5 nM]. In contrast, palbociclib has comparable activity against CDK4 [IC_50_ = 9–11 nM] and CDK6 [IC_50_ = 9–11 nM]^11^. Abemaciclib also inhibits CDK2/Cyclin A/E and CDK1/Cyclin B. In addition to cell cycle blockade, CDK4/6i can also induce a senescence-like state, promote epigenetic remodelling, autophagy, blockade of oncogenic signalling networks and promote tumor immunogenicity^9,10^. Preclinically, ribociclib, abemaciclib, and palbociclib treatment can induce differential transcriptional and proteomic responses in breast cancer cells, implying a complex pharmacodynamic activity^12^.

In estrogen receptor–positive (ER + ) and human epidermal growth factor receptor-2–negative (HER2–) advanced breast cancers, ribociclib, palbocilib, and abemaciclib significantly improve survival outcomes in patients^13^. In the first-line post-menopausal setting, all three CDK4/6 inhibitors in combination with an aromatase inhibitor (AI), compared to placebo, significantly improved progression-free survival (PFS)^14–17^. However, significant median overall survival (mOS) benefit was shown for ribociclib compared to placebo (63.9 months vs. 51.4 months)^18^ but not for abemaciclib (mOS was 66.8 vs. 53.7)^19^ or Palbociclib (53.9 months vs. 51.2 months)^20^. Most patients receiving chronic CDK4/6i therapy eventually progress. Development of acquired or intrinsic resistance to CDK4/6i is an emerging clinical challenge.

We hypothesized that the development of resistance to ribociclib, abemaciclib, and palbociclib in various ER+ /HER2− breast cancer cells is complex. Preclinically, we developed ribociclib, abemaciclib or palbociclib-resistant ER+ breast cancer models and investigated the transcriptomic alterations in the CDK4/6i-resistant setting. We then searched for cell cycle-regulating kinases that could be targeted. We observed upregulation of aurora kinase B (*AukB*) and polo-like kinase 1 (*PLK1*) in resistant cells. Resistant cells were sensitive to volasertib (a PLK1 inhibitor) and barasertib (an AukB inhibitor) therapy, which is associated with G2/M cell cycle arrest and increased apoptosis.

## Materials and methods

### Pre-clinical study

#### Cell lines and tissue culture

T47D (ER + ) breast cancer cell lines were purchased from (Signosis, SL-0002) and MCF7 ER+ breast cancer cell lines were purchased from the American Type Culture Collection (ATCC, Manassas, USA). . T47D cells were grown in RPMI (R8758, Merck, UK) and MCF7 cells were grown in DMEM. All media were supplemented with 10% fetal bovine serum (F4135; Merck, UK) and 1% penicillin–streptomycin (P4333, Merck, UK).

#### Compounds and reagents

Ribociclib, Abemaciclib and Palbociclib were purchased from Selleckchem (UK). The compounds were suspended in 100% *v/v* dimethyl sulfoxide (DMSO) (276855-250ML, Sigma Aldrich, UK) at 10 mM and stored at − 80 °C. Cisplatin was kindly provided as a 3.3 mM solution by the pharmacy at Nottingham University City Hospital (Nottingham, UK) and stored at room temperature. 17β–Estradiol was purchased from Sigma.

#### Generation of ribocilcib, abemaciclib and palbociclib resistant T47D and MCF7 cell lines

**Fig. 1:**
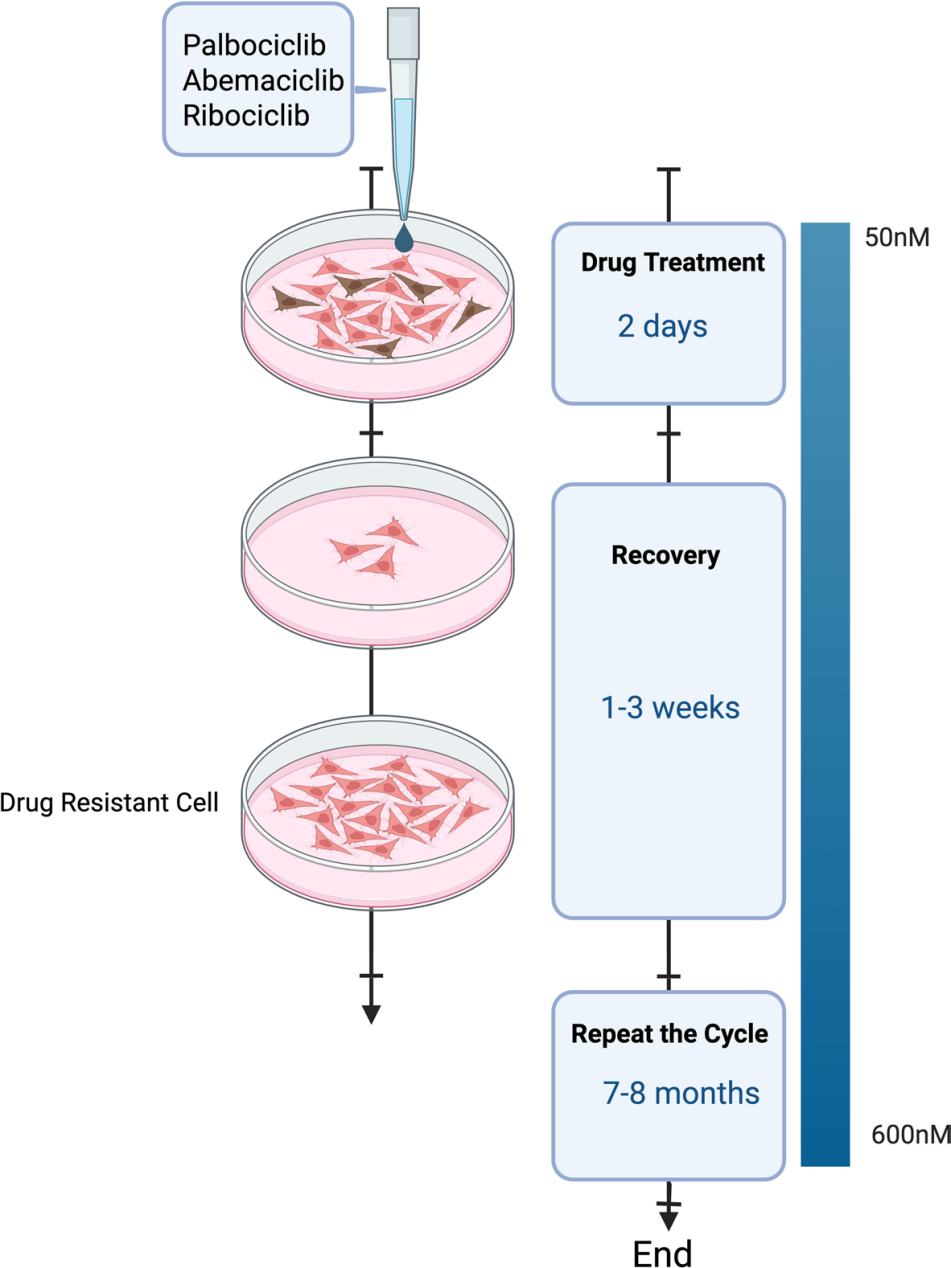
Generation of ribocilcib, abemaciclib and palbociclib resistant T47D and MCF7 cell lines: T47D and MCF7 was treated with increasing doses of ribociclib, abemaciclib, or palbociclib (50–600 nM). Please see methods section for full details. The figure was created in BioRender [Algethami, M. (2025) https://BioRender.com/bns6ond].

T47D and MCF7 was treated with increasing doses of ribociclib, abemaciclib, or palbociclib (50–600 nM). At each dose level, T47D and MCF7 were maintained for three generations. Ribociclib (T47D-RR, MCF7-RR)-, abemaciclib (T47D-RA, MCF7-RA)-, and palbociclib (T47D-RP, MCF7-RP)-resistant cell lines were established from parenteral T47D and MCF7 cells over a period of eight months (Fig. 1).

#### Generation of 3D spheroids

A total of 4 × 10^4^ cells per well were plated in ultra-low attachment 6-well plates in Promocell serum-free tumor sphere medium (C-28070). Cells were then topped off with fresh medium every three days until spheroid structures were formed. The spheroids were treated with Ribociclib, Abemaciclib and Palbociclib inhibitors for 48 h.

#### Confocal microscopy

Spheroids were fixed with 4% paraformaldehyde for 30 min. After fixation, the spheroids were washed carefully, and a solution in PBS containing 2 µM Calcein AM and 1 µM Ethidium Homodimer-1 was added to the 3D structures. The spheroids were incubated in the dark at room temperature for 30 min. Imaging was performed using a Leica SP8 confocal laser scanning microscope (CLSM). The ImageJ software (version 1.54 h) (https://imagej.net/ij/download.html) was used to calculate the spheroid diameter. The mean of the three diagonal diameters was taken as the diameter of each spheroid. A minimum of 10 spheroids were identified.

#### Cell doubling time

1 × 10^5^ cells were seeded in 6 well plates and incubated for 24, 48, 72 h for MCF7 cells and 40, 80 120 h for T47D cells. The cells were trypsinized and counted using a cell drop automated cell counter.

#### Western blot analysis

Cells were harvested and lysed in RIPA buffer (R0278, Sigma-Aldrich) with the addition of protease cocktail inhibitor (P8348, Sigma, UK), phosphatase inhibitor cocktail 2 (P5726, Sigma, UK), and phosphatase inhibitor cocktail 3 (P0044, Sigma), and stored at − 20 °C. Proteins were quantified using a BCA Protein Assay Kit (23,225, Thermofisher, UK). The samples were run on SDS-bolt gel (4–12%) bis–tris. Membranes were incubated with the following primary antibodies overnight at 4 °C as follows: Anti-CDK2 (1:800, ab32147 Abcam), Anti-CDK4 (1:750, ab108357 Abcam), Anti-CDK6 (1:750, ab124821 Abcam), Anti-Cyclin D1(1:150, ab16663 Abcam), anti-ER-alpha (1:1000, ab32063 Abcam), and Anti-HER2 (1:1000, ab134182 Abcam). Anti-β-actin (1:5000, ab8226 Abcam) was used as a loading control and incubated at room temperature for 1 h. Membranes were then washed and incubated with infrared dye-labelled secondary antibodies (LiCor) [IRDye 800CW Donkey Anti-Rabbit IgG (926–32,213) and IRDye 680CW Donkey Anti-Mouse IgG (926–68072)] at a dilution of 1:10,000 for 1 h. Membranes were scanned with a LiCor Odyssey machine (700 and 800 nm) to determine protein levels.

#### Luciferase assay in T47D

3 × 10^4^ cells per well) were seeded in 96 well white clear bottom plates and incubated overnight at 37 °C in a 5% CO2 atmosphere. After 24 h, 0.1 nM of the inducing reagents (17β –Estradiol) were added to the cells and incubated for 24 h. The cells were then washed with phosphate-buffered saline (PBS). 1 × lysis buffer (Signosis LS-001) was added to each well and incubated for 15 min at room temperature. The luciferase substrate (100 µL; Signosis LUC015) was added to each well and gently pipetted up and down. The plates were then read using a luminometer (FluoroSTAR plate reader).

#### Real-time PCR

RNA was extracted using an RNeasy Mini Kit (74,104, Qiagen) and quantified using a Nanodrop 2000c (Thermo Fisher Scientific). cDNA synthesis was performed using an RT2 First Strand Kit (330,404, Qiagen, Germany). Real-time PCR was performed on an Applied Biosystems 75,000 FAST Cycler.

#### Functional studies

1 × 10^6^ cells per well were seeded in 6- well plates and incubated overnight at 37 °C in a 5% CO2 atmosphere. After 24 h, 600 nM ribociclib, abemaciclib, or palbociclib was added to cells and incubated for 48 h. Cells then were collected by trypsinization, washed with ice cold PBS, and fixed in 70% ethanol for 1 h at 4 °C. After removal of the fixative solution by centrifugation, for cell cycle analysis, cells were treated with 20 mg/ml RNase A (12,091,021, Invitrogen), and then 10 mg/ml Propidium Iodide (P4170, Sigma Aldrich) was added to determine the cell cycle distribution. The samples were analyzed on a Beckman Coulter FC500 flow cytometer using a 488 nm laser for excitation and emission data for PI collected using a 620 nm bandpass filter (FL3) and a 525 nm bandpass filter (FL1) for FITC-anti-phospho-histone H2A.X. For the Apoptosis assay, cells were analyzed using an Annexin V detection kit (556,547, BD Biosciences). Briefly, cells were trypsinized, washed with PBS, and the cellular pellet was resuspended in Annexin Binding Buffer (1x). Next, 1 µM FITC Annexin V and 1 µM Propidium Iodide were added to the cells. After incubation, 300 ml of Annexin Binding Buffer (1x) was added to each tube. The samples were analyzed on a Beckman Coulter FC500 flow cytometer. Data were analyzed using Weasel software (version 3.0.2) ImageJ.

#### RNA seq analysis

See supplementary methods for full details.

### Clinical study

#### CDK4, CDK6 and p53 protein expression in ER+ invasive breast cancer

This study was performed in a large series of 1005 invasive breast cancer cases treated at Nottingham University Hospitals (NUH) between 1986 and 2006. All patients were treated at a single institution and investigated in a wide range of biomarker studies. Supplementary Table 2 summarizes the patient demographics. The patients underwent standard surgery (mastectomy or wide local excision) with radiotherapy. Prior to 1989, the patients did not receive systemic adjuvant treatment (AT). After 1989, AT was scheduled based on the prognostic and predictive factor status, including Nottingham Prognostic Index (NPI), estrogen receptor-α (ER-α) status, and menopausal status. Patients with NPI scores < 3.4 (low risk) did not receive AT. In pre-menopausal patients with NPI scores ≥ 3.4 (high risk), classical Cyclophosphamide, Methotrexate, and 5-Flourouracil (CMF) chemotherapy was administered; patients with ER-α-positive tumors were also offered HT. Postmenopausal patients with NPI scores of ≥ 3.4 and ER-α positivity were offered HT, while ER-α-negative patients received classical CMF chemotherapy. The median follow-up period was 111 months (range: 1–233 months). Survival data, including breast cancer-specific survival (BCSS) and the development of locoregional and distant metastases (DM), were prospectively maintained. Breast cancer-specific survival (BCSS) was defined as the number of months from diagnosis to the occurrence of BC deaths. DM-free survival was defined as the number of months from the diagnosis to the occurrence of DM relapse. Survival was censored if the patient was still alive at the time of analysis, lost to follow-up, or died of other causes.

This study was approved by the ethics Committee of the Northwest – Greater Manchester Central Research Ethics Committee under the title Nottingham Health Science Biobank (NHSB), reference number 15/NW/0685. Informed consent was obtained from all individuals prior to surgery to use their tissue materials in the research. All the samples used in this study were pseudoanonymized, collected prior to 2006, and stored in compliance with the UK Human Tissue Act. Informed consent was obtained from the patient. The Reporting Recommendations for Tumor Marker Prognostic Studies (REMARK) criteria recommended by McShane et al.^21^ were followed throughout this study. All methods were performed in accordance with the relevant guidelines and regulations.

#### Tissue microarray (TMA) and immunohistochemistry (IHC)

Tissue Microarrays (TMAs) were placed in neutral-buffered formalin to prevent diffusion issues and embedded into the paraffin block. Staining was conducted on 4 μm thick. Immunohistochemical staining was conducted using the Novolink Max Polymer Detection System (RE7280-K: 1250 tests) and Leica Bond Primary Antibody Diluent (AR9352), each used according to the manufacturer’s instructions (Leica Microsystems). Tissue slides were deparaffinized with xylene and then rehydrated using five decreasing concentrations of alcohol (100%, 90%, 70%, 50%, and 30%) for two minutes each. Pre-treatment antigen retrieval was performed on the TMA sections using sodium citrate buffer (pH 6.0) and heated for 20 min at 95 °C in a microwave (Whirlpool JT359 Jet Chef 1000W). Anti-CDK4 monoclonal antibody (DCS-31) (Invitrogen, UK) and Anti CDK6 monoclonal antibody (SD20-50) (Invitrogen, UK) were diluted at 1:20 and 1:15, respectively, in Leica antibody diluent (RE AR9352, Leica, Biosystems, UK) and incubated for 60 min at room temperature. Rabbit polyclonal anti-PLK1 antibody (ab109777, Abcam, UK) was diluted 1:250 in Leica antibody diluent (RE AR9352, Leica, Biosystems, UK) and incubated for 30 min at room temperature. Normal kidney tissue was used as a positive control for CDK4, normal liver tissue was used as a positive control for CDK6.Negative control was obtained by omitting the primary antibodies. The sections were counterstained with hematoxylin. The sections were counterstained with hematoxylin.

#### Evaluation of immunohistochemical staining

Whole-field inspection of the core was scored, and the subcellular localization of each marker was identified (nuclear, cytoplasmic, and cell membrane). The intensities of subcellular compartments were assessed and grouped as follows: 0, no staining, 1 = weak staining, 2 = moderate staining, 3 = strong staining. The percentage of tumor cells in each category was estimated (0–100%). The histochemical score (H-score) (range, 0–300) was calculated by multiplying the intensity and percentage of staining. X-tile bioinformatics software (version 3.6.1 (School of Medicine, Yale University, New Haven, CT, USA) was used^22^ to categorise CDK4 and CDK6 H-scores into low and high expression, respectively. An H-score of 110 was the best cutoff for nuclear expression of CDK4, and an H-score of 80 was the best cutoff for nuclear expression of CDK6. Immunostaining for p53 showed nuclear expression, and its score was evaluated using the H-score. A 10% cut-off was used as the optimal cut-off of categorisation of p53 expression into negative (wild-type) and positive (mutant) tumours based on X tile.

#### AurkB and PLK1 transcripts in breast cancers

We evaluated the clinicopathological significance of *AurkB* and *PLK1* transcripts in publicly available clinical breast cancer datasets (https://bcgenex.ico.unicancer.fr/BC-GEM/GEM-Accueil.php?js=1)^23,24^.

### Statistical analysis

Graphical representation and statistical analyses were performed using GraphPad Prism 9 (GraphPad Software, La Jolla, CA, USA) (https://www.graphpad.com/). The Student’s t-test was performed to compare the two groups. One-way analysis of variance (ANOVA) was performed to compare more than two groups. Two-way ANOVA was used to analyze two variables: Annexin V analysis and cell cycle analysis. All experiments are expressed as the mean ± standard deviation S.D. of three independent experiments. *p*-value < 0.05 = *, *p*-value < 0.01 = **, *p*-value < 0.001 = *** & *p*-value < 0.0001 = ****. In the clinical study, the association of clinical and pathological parameters using categorized data was examined using the chi-squared test. All tests were 2-tailed. Survival rates were determined using the Kaplan–Meier method and compared using the log-rank test. All analyses were conducted using Statistical Package for the Social Sciences (SPSS, version 22, Chicago, IL, USA) software for Windows. Statistical significance was set at *p* < 0.05.

## Results

### Generation of CDK4/6i-resistant breast cancer cells (Fig. 1)

**Fig. 2:**
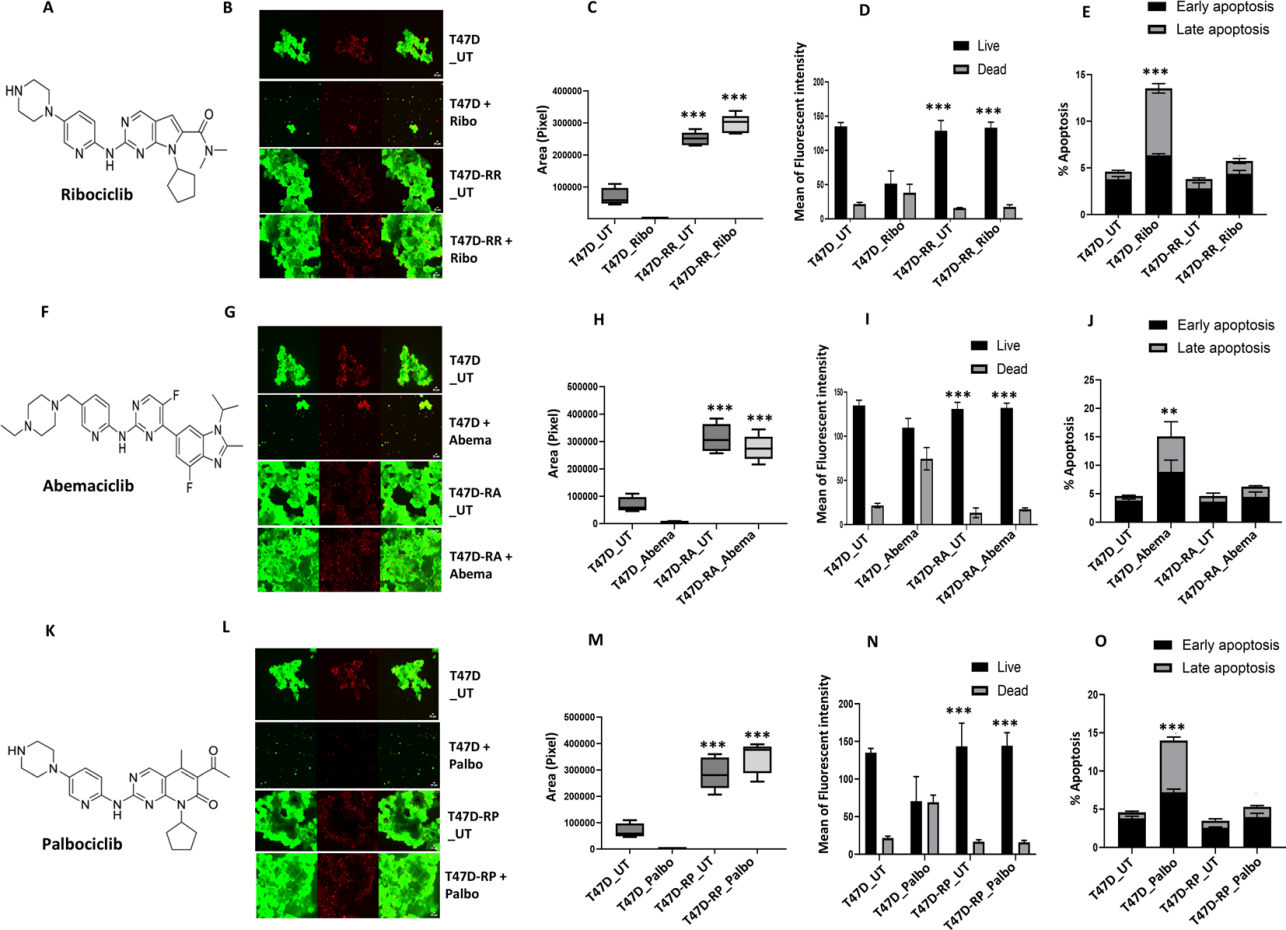
Evaluation of CDK4/6i resistant T47D cells. (**A**) Chemical structure of ribociclib. (**B**) T47D control and ribociclib resistant spheroids untreated (UT) or treated with 600 nM of ribociclib. (**C**) Spheroid size in control and resistant spheroids untreated (UT) or treated with 600 nM of ribociclib. (**D**) Proportion of living and dead cells in control and resistant spheroids untreated (UT) or treated with 600 nM of ribociclib. (**E**) % of apoptotic cells in untreated (UT) or treated with 600 nM of ribociclib. (**F**) Chemical structure of abemaciclib. (**G**) T47D control and abemaciclib resistant spheroids untreated (UT) or treated with 600 nM of abemaciclib. (**H**) Spheroid size in control and resistant spheroids untreated (UT) or treated with 600 nM of abemaciclib. (**I**) Proportion of living and dead cells in control and resistant spheroids untreated (UT) or treated with 600 nM of abemaciclib. (**J**) % of apoptotic cells in untreated (UT) or treated with 600 nM of abemaciclib. (**K**) Chemical structure of palbociclib. (**L**) T47D control and palbociclib resistant spheroids untreated (UT) or treated with 600 nM of palbociclib. (**M**) Spheroid size in control and resistant spheroids untreated (UT) or treated with 600 nM of palbociclib. (**N**) Proportion of living and dead cells in control and resistant spheroids untreated (UT) or treated with 600 nM of palbociclib. (**O**) % of apoptotic cells in untreated (UT) or treated with 600 nM of palbociclib. All the comparative analyses were between two sets of groups: Control_UT versus R_UT and Control_T versus R_T. ‘*’- *p* ≤ 0.05, ‘**’- *p* ≤ 0.01, ‘***’- *p* ≤ 0.001. All figures are representative of 3 or more experiments. Error bars represent standard error of mean between experiments.

T47D and MCF7 (ER+ /HER2 −) were chronically treated with increasing doses of ribociclib (R; Fig. 2A), abemaciclib (A; Fig. 2F), or palbociclib (P; Fig. 2K) over 8 months (0–600 nM). At each dose (0, 50, 100, 200, 300, 400, 500, and 600 nM), MCF7 and T47D cells were maintained for at least three generations to obtain CDK4/6i-resistant cell lines (T47DrR, T47DrA, T47DrP, MCF7rR, MCF7rA, and MCF7rP).

To confirm the resistant phenotype of these cell line models, we began by testing their spheroid-forming ability and measuring apoptosis in response to their respective CDK4/6i. Compared to T47D control spheroids, T47DrR (Fig. 2B–E), T47DrA (Fig. 2G–J), and T47DrP (Fig. 2L–O) spheroids were resistant to 600 nM ribociclib, abemaciclib, and palbociclib treatment, respectively, showing enhanced spheroid formation (Fig. 2C,H,M), lower levels of apoptosis (Fig. 2D,E,I,J,N,O), and increased clonogenicity (Supplementary Fig. 1). We then examined cross-resistance to the other CDK4/6i. T47DrR spheroids were cross-resistant to 600 nM abemaciclib but remained sensitive to palbociclib treatment (Supplementary Fig. 2A–C). T47DrA was cross resistant to ribociclib and palbociclib treatment (Supplementary Fig. 2D–F) and T47DrP was cross resistant to ribociclib and abemaciclib treatment (Supplementary Fig. 2G,H,I).

**Fig. 3:**
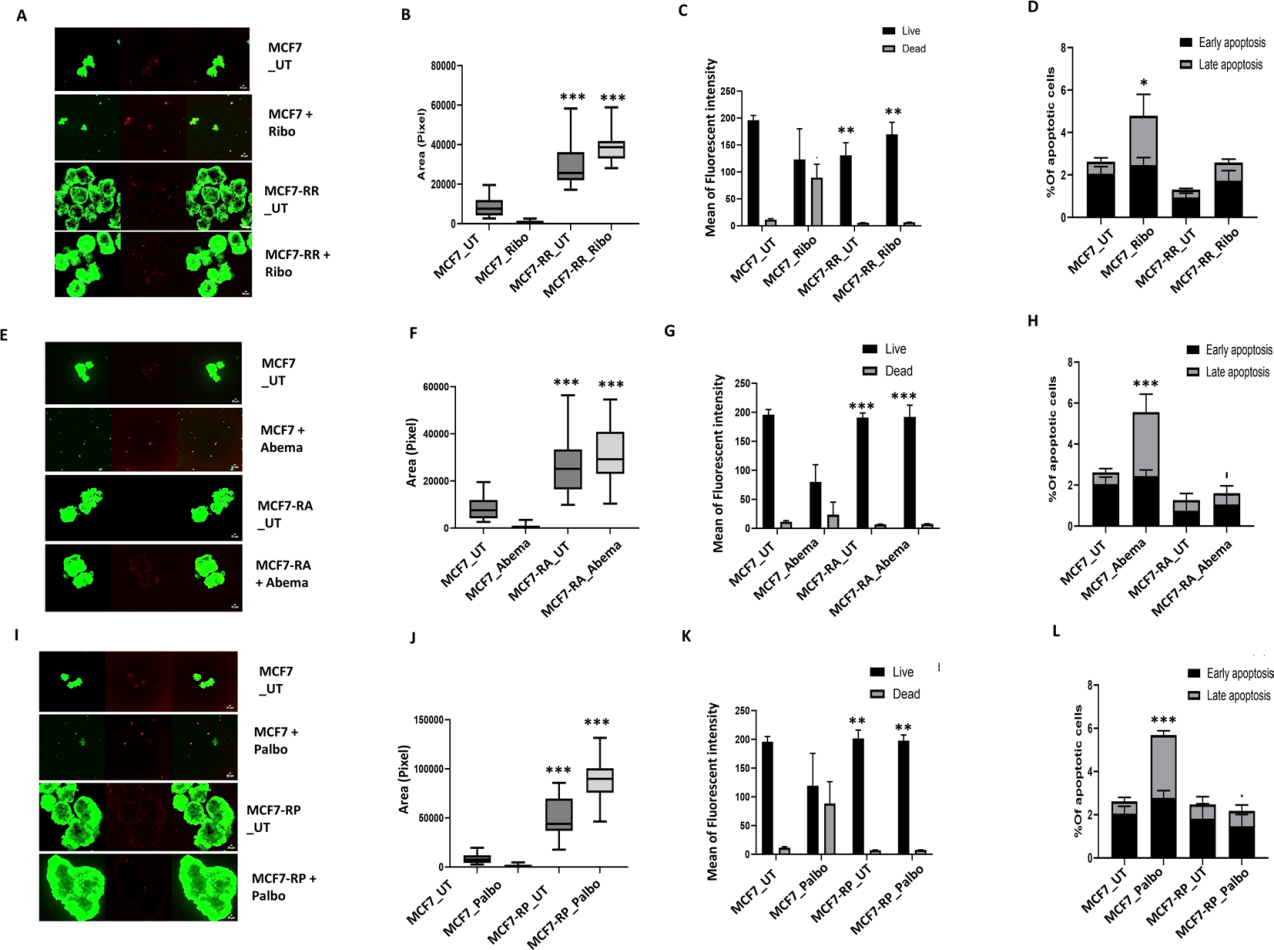
Evaluation of CDK4/6i resistant MCF7 cells. (**A**) MCF7 control and ribociclib resistant spheroids untreated (UT) or treated with 600 nM of ribociclib. (**B**) Spheroid size in control and resistant spheroids untreated (UT) or treated with 600 nM of ribociclib. (**C**) Proportion of living and dead cells in control and resistant spheroids untreated (UT) or treated with 600 nM of ribociclib. (**D**) % of apoptotic cells in untreated (UT) or treated with 600 nM of ribociclib. (**E**) MCF7 control and abemaciclib resistant spheroids untreated (UT) or treated with 600 nM of abemaciclib. (**F**) Spheroid size in control and resistant spheroids untreated (UT) or treated with 600 nM of abemaciclib. (**G**) Proportion of living and dead cells in control and resistant spheroids untreated (UT) or treated with 600 nM of abemaciclib. (**H**) % of apoptotic cells in untreated (UT) or treated with 600 nM of abemaciclib. (**I**) MCF7 control and palbociclib resistant spheroids untreated (UT) or treated with 600 nM of palbociclib. (**J**) Spheroid size in control and resistant spheroids untreated (UT) or treated with 600 nM of palbociclib. (**K**) Proportion of living and dead cells in control and resistant spheroids untreated (UT) or treated with 600 nM of palbociclib. (**L**) % of apoptotic cells in untreated (UT) or treated with 600 nM of palbociclib. All the comparative analyses were between two sets of groups: Control_UT versus R_UT and Control_T versus R_T. ‘*’- *p* ≤ 0.05, ‘**’- *p* ≤ 0.01, ‘***’- *p* ≤ 0.001. All figures are representative of 3 or more experiments. Error bars represent standard error of mean between experiments.

Compared to MCF7 control spheroids, MCF7rR (Fig. 3A–D), MCF7rA (Fig. 3E–H) and MCF7rP (Fig. 3I–L) spheroids also conferred resistance to 600 nM of ribociclib, abemaciclib or palbociclib treatment respectively, through increased spheroid formation, reduced cell death and increased clonogenic capacity (Supplementary Fig. 1). Cross-resistance to the other CDK4/6i was also evident in the resistant MCF7 spheroids (MCF7rR; Supplementary Fig. 3A–C; MCF7rA, Supplementary Fig. 3D–F and Supplementary Fig. 3G–I).

### CDK4/6i-resistant cells manifest an aggressive phenotype

**Fig. 4:**
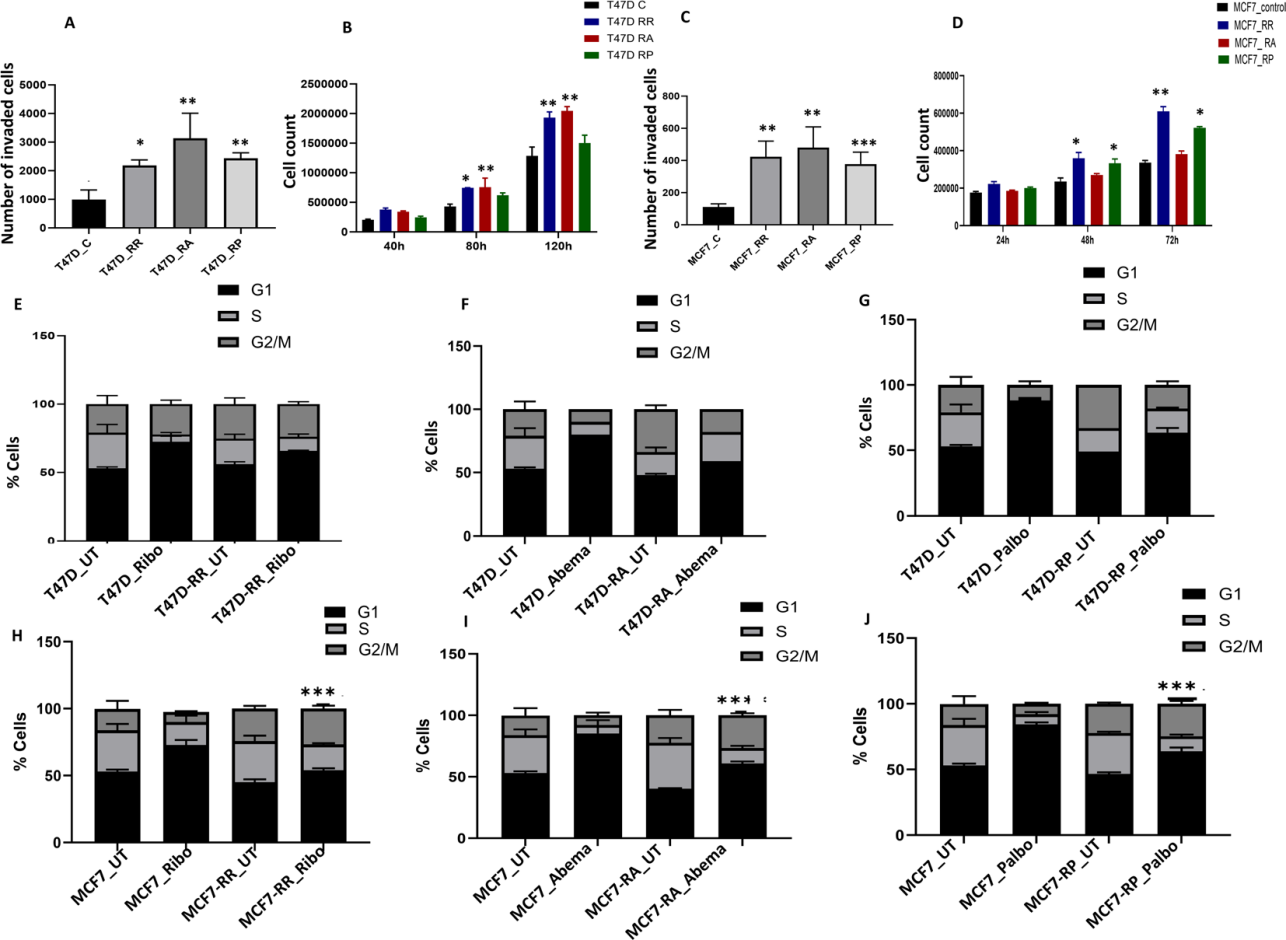
CDK4/6i resistant cells have aggressive phenotype and bypass G1/S. (**A**) Invasion assay in T47D control and resistant cells. (**B**) Cell doubling times in T47D control and resistant cells. (**C**) Invasion assay in MCF7 control and resistant cells. (**D**) Cell doubling times in MCF7 control and resistant cells. (**E**) Cell cycle progression in T47D control and ribociclib resistant cells untreated (UT) or treated with ribociclib. (**F**) Cell cycle progression in T47D control and abemaciclib resistant cells untreated (UT) or treated with abemaciclib. (**G**) Cell cycle progression in T47D control and palbociclib resistant cells untreated (UT) or treated with palbociclib. (**H**) Cell cycle progression in MCF7 control and ribociclib resistant cells untreated (UT) or treated with ribociclib. (**I**) Cell cycle progression in MCF7 control and abemaciclib resistant cells untreated (UT) or treated with abemaciclib. (**J**) Cell cycle progression in MCF7 control and palbociclib resistant cells untreated (UT) or treated with palbociclib. The comparative analyses for 3A were between T47D_C and T47D resistant cells, and for 3C, they were between MCF7_C and MCF7 resistant cells. The comparative analysis for 3B and 3D were between control and resistant cells for each time point. For the cell cycle progression (3H, 3I, and 3 J), the comparisons were between G1 phase Control_T and G1 phase R_T**.**‘*’- *p* ≤ 0.05, ‘**’- *p* ≤ 0.01, ‘***’- *p* ≤ 0.001. All figures are representative of 3 or more experiments. Error bars represent standard error of mean between experiments.

The spheroid-forming 2ability of T47DrR (Fig. 2C), T47DrA (Fig. 2H), and T47DrP (Fig. 1M) cells was significantly higher than that of the T47D control cells. Moreover, T47D resistant cells were not only resistant to apoptosis (Fig. 2E,J,O) but also showed significantly increased invasion (Fig. 4A) and proliferation (Fig. 4B) compared to T47D controls. Similarly, MCF7 resistant cells also manifested an aggressive phenotype, including increased spheroid-forming ability (Fig. 3B,F,J), resistance to apoptosis (Fig. 3D,H,L), high invasiveness (Fig. 4C), and enhanced proliferative capacity (Fig. 4D), compared to MCF7 control cells.

### Resistant cells progress through G1/S despite CDK4/6i therapy

Cell cycle progression was then analyzed by FACS. Following treatment with 600 nM ribociclib (Fig. 4E), abemaciclib (Fig. 4F), or palbociclib (Fig. 4G), T47D control cells were arrested at G1. However, despite 600 nM treatment with ribociclib, abemaciclib, or palbociclib, T47D resistant cells (Fig. 4E–G) showed a reduced fraction of cells in G1 phase and progressed through the S and G2/M phases of the cell cycle. Despite CDK4/6I treatment, overall MCF7 resistant cells show a reduced fraction cells in G1 but progress through S and G2/M phases (Fig. 4H–J) (the percentage of cells in various stages of the cell cycle is shown in Supplementary Table 1). These data provide evidence that resistant cells may have evolved bypass mechanisms to progress through G1.

### Cyclin D1, CDK2, CDK4, CDK6, ER and HER2 protein expression in CDK4/6i-resistant cells compared to controls

Whole cell lysates from control cells (T47D and MCF7) and resistant cells (T47DrR, T47DrA, T47DrP, MCF7rR, MCF7rA, and MCF7rP) were analyzed for CDK2/4/6 and cyclinD1 (Supplementary Fig. 4). Increased CDK4 expression was observed in T47DrR (Supplementary Fig. 4A,B) and MCF7rR (Supplementary Fig. 4I,J). Higher CDK6 expression levels were observed in T47DrR (Supplementary Fig. 4C,D), T47DrA (Supplementary Fig. 4C,D) and MCF7rP cells (Supplementary Fig. 4K,L). Increased cyclin D1 expression was observed in MCF7rP cells (Supplementary Fig. 4,4N). Higher levels of CDK2 expression were observed in T47DrP cells (Supplementary Fig. 4G,H) and MCF7rR cells (Supplementary Fig. 4O,P). Lower ER expression was observed in T47DrA, T47DrP, MCF7rA, and MCF7rP (Supplementary Fig. 5A,B,E,F). HER2 overexpression was observed only in MCF7rP cells (Supplementary Fig. 5C,D,G,H) compared to that in the controls. These data provide evidence for a complex pattern of expression that may provide a selective survival advantage for CDK4/6i-resistant cells compared to sensitive cells.

### Global transcriptomic analysis

To investigate dysregulation at the global genomic level, we performed a transcriptomic analysis. We hypothesized that the development of resistance to CDK4/6i is likely multifactorial and may involve dysregulation of genes involved in pro-proliferative signalling, evasion of growth suppression, activation of invasion, metastasis, angiogenesis, immune evasion, and/or altered cellular energetics. As ribociclib, abemaciclib, and palbociclib are distinct chemical entities, we speculated that resistance mechanisms would include unique patterns of dysregulated gene expression. Moreover, preclinically ribociclib, abemaciclib, and palbociclib treatment have previously been shown to induce differential transcriptional responses in breast cancer cells, implying a complex pharmacodynamic activity^12^.

**Fig. 5:**
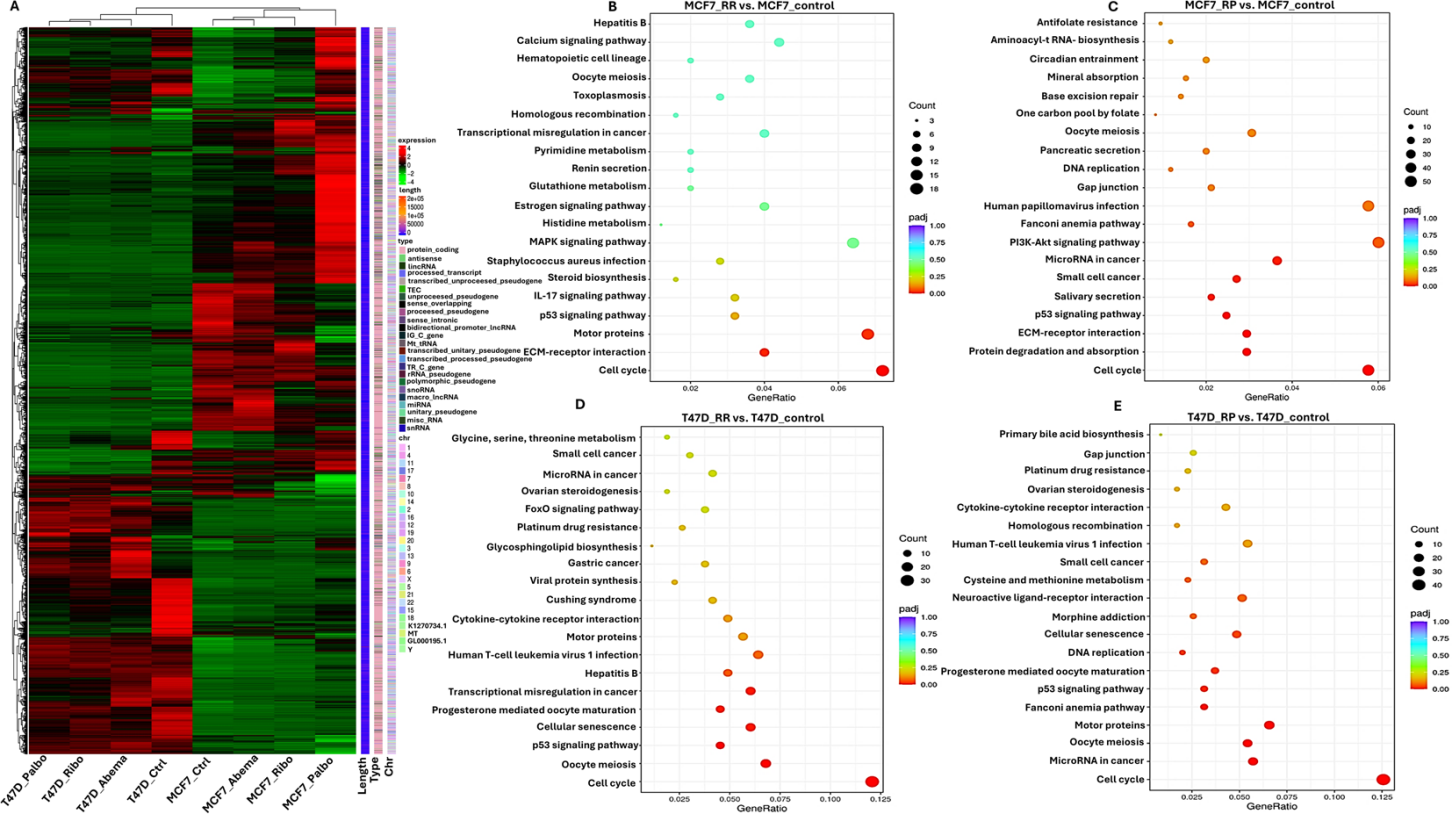
RNA sequencing analysis. (**A**) Hierarchical clustering analysis (HCA) in T47D or MCF7 control and resistant cells. We used the mainstream hierarchical clustering to cluster the fpkm values of genes and homogenized the row (Z-score). The genes or samples with similar expression patterns in the heat map will be gathered together. The colour in each grid reflects not the gene expression value, but the value obtained after homogenizing the expression data rows (generally between—2 and 2). Therefore, the colours in the heat map can only be compared horizontally (the expression of the same gene in different samples), but not vertically (the same sample). There are not only inter group clustering, but also inter sample clustering. (**B**) Kyoto Encyclopedia of Genes and Genomes (KEGG) analysis in MCF7_RR compared to MCF7_control cells. The most significant 20 KEGG pathways were selected for display. The abscissa is the ratio of the number of differential genes linked with the KEGG pathway to the total number of differential genes. The ordinate is KEGG Pathway. The size of a point represents the number of genes annotated to a specific KEGG pathway. The colour from red to purple represents the significant level of the enrichment. (**C**) KEGG analysis in MCF7_RP compared to MCF7_control cells. (**D**) KEGG analysis in T47D_RR compared to T47D_control cells. (**E**) KEGG analysis in T47D_RP compared to T47D_control cells. See supplementary methods 2 for full details.

We conducted a transcriptomic analysis of MCF7_control versus MCF7 resistant cells and of T47D_control versus T47D resistant cells. All experiments were completed in triplicates. Principal component analysis (PCA) of the gene expression value (FPKM) showed that the two cell lines (MCF7-p53 WT and T47D-p53 mutated) had distinct groupings (Supplementary Fig. 6A). We then evaluated genes that were uniquely expressed within each resistant cell line compared to the parental cells. (Venn diagrams are shown in Supplementary Fig. 6B–G). A differential gene expression analysis was performed. Volcano plots show the overall distribution of differentially expressed genes in MCF7_control versus MCF7 resistant cells, and in T47D_control versus T47D resistant cells (Supplementary Fig. 7A-F; supplementary data 1). Hierarchical clustering analysis (HCA) (Fig. 5A) revealed distinct patterns of gene expression in T47D_control and MCF7_control cells, implying that although both cell lines were ER+ , they exhibited distinct basal gene expression patterns (Fig. 5A). Moreover, HCA analysis between MCF7_control and MCF7 resistant cells showed a greater distinctive pattern of expression in MCF7rP compared to the control and other resistant cells, whereas the gene expression pattern in the T47D resistant cells was similar to that in the parental cells (Fig. 5A). This implies that each CDK4/6i-resistant cell line had a specific altered gene expression signature. Functional analysis using clusterProfiler software [(version 3.10.1) (https://bioconductor.org/packages/release/bioc/html/clusterProfiler.html)] was performed to explore biological functions or pathways that were significantly associated with differentially expressed genes in CDK4/6i-resistant cell lines compared to controls. The full data are shown in Supplementary data 2–13. Because the interactions of multiple genes may be involved in certain biological functions, Kyoto Encyclopedia of Genes and Genomes (KEGG) analysis was performed. KEGG pathways with *p* value (adj) < 0.05 were considered as significant enrichment. The 20 most significant KEGG pathways are shown for MCF7_control versus MCF7 resistant cells (Fig. 5B,C; supplementary 7G respectively, Supplementary data 16, 17 and 20) and T47D_control versus T47D resistant cells (Fig. 5D–E; Supplementary Fig. 7H, respectively; Supplementary data 18–21). Enrichment of the cell cycle (Fig. 5A–E and Supplementary Fig. 7G–H) and p53 signalling (Fig. 5A–E and Supplementary Fig. 7G–H) were frequently observed. Gene ontology (GO) enrichment analysis, where GO terms with *p*adj < 0.05, were considered significant enrichment. The most significant 30 GO Terms are displayed for MCF7_control versus MCF7 resistant cells, and T47D_control versus T47D resistant cells are shown in Supplementary Fig. 8A–F. Genes involved in chromosomal segregation, sister chromatid segregation, and mitotic nuclear division were frequently observed (Supplementary Fig. 8A-F, Supplementary data 22–27). Reactome Enrichment Analysis was performed to confirm the biological pathways identified using KEGG. The 20 most significant Reactome pathways (*p*adj < 0.05) are displayed for MCF7_control versus MCF7 resistant cells, and T47D_control versus T47D resistant cells are shown in Supplementary Fig. 9A–F. Enrichment of the pathways involved in mitosis was consistently observed (Supplementary Fig. 9A–F, Supplementary data 28–33).

ER signalling pathway in control and CDK4/6i-resistant cells: As shown in Supplementary Fig. 5A,B,E,F, downregulation of ER was observed in T47DrA, T47DrP, MCF7rA, and MCF7rP cells. The T47D control cells used in the current study were an ER-responsive luciferase reporter T47D stable cell line (Signosis, SL-0002) that stably expresses the firefly luciferase reporter gene under the control of the ER response element. Therefore, we used this system to evaluate, irrespective of ER expression, the activation of the ER signalling pathway in T47D control cells and compared it to that in T47DrA, T47DrP, and T47DrR cells. As expected, T47DrR cells with robust ER expression showed increased luciferase activity (Supplementary Fig. 10A). Surprisingly, despite reduced ER expression, a significant increase in luciferase activity was observed in T47DrA (Supplementary Fig. 10B) and T47DrP (Supplementary Fig. 10C) cells, indicating enhanced ER signalling. Gene set enrichment (GSEA) analysis also showed ER signalling enrichment in T47DrR (Supplementary Fig. 10D, Supplementary data 34), T47DrA (Supplementary Fig. 10E, Supplementary data 35).

Enrichment of p53 signalling in CDK4/6i resistant cells: KEGG analysis revealed p53 signalling enrichment in CDK4/6I resistant cells (Fig. 5A–E, Supplementary Fig. 7G and 7H, Supplementary data 16, 17, 18., 19, 20 and 21) a feature that was also observed in the GSEA analysis of MCF7rR, MCF7rP, and MCF7rA cells (Supplementary Fig. 10F–H, Supplementary data 36–38). To understand the prognostic significance of CDK4 or CDK6 expression in wild type p53 or mutant p53, we immunohistochemically evaluated a clinical cohort of 1055 CDK4/6i-naïve breast cancers. Patient demographics are summarized in Supplementary Table 2. We observed the nuclear and cytoplasmic expression of CDK4 and CDK6 (Supplementary Fig. 11A,D).

In p53 wild-type tumors, CDK4 expression was significantly associated with tumor grade (*p* = 0.008), tumor type (*p* < 0.0001), and molecular subtype (*p* = 0.037) (Supplementary Table 3). (Supplementary Table 3). In p53-mutated tumors, CDK4 expression was associated with tumor type (*p* = 0.009) and showed a borderline association with tumor grade (*p* = 0.05) (Supplementary Table 4). In p53_wildtype and p53_mutated tumors, CDK4 expression did not influence survival (Supplementary Fig. 11,C).

In p53 wild-type tumors, CDK6 expression was significantly associated with age (*p* = 0.002), menopausal status (*p* = 0.035), tumor grade (*p* < 0.0001), histologic subtype (*p* < 0.0001), molecular subtype (*p* = 0.017), NPI (*p* = 0.039), and Ki67 proliferative index (*p* = 0.03) (Supplementary Table 5). In p53-mutated tumors, CDK6 expression was significantly associated with tumor size (*p* = 0.047), tumor grade (*p* = 0.008), histologic subtype (*p* < 0.001), molecular subtype (*p* = 0.017), NPI (*p* = 0.039), and Ki67 proliferative index (*p* = 0.03) (Supplementary Table 6). Additionally, in p53-mutated tumors, higher CDK6 expression was significantly associated with poorer breast cancer-specific survival (Supplementary Fig. 11E), whereas no such association was observed in p53 wild-type tumors (Supplementary Fig. 11F).

In a multivariate analysis, CDK6 expression showed independent association with patient outcomes independent of other confounders in p53 mutant tumours (*p* = 0.044) (Supplementary Table 7).

### Cell cycle dysregulation in CDK4/6i-resistant cells leads to susceptibility to inhibition of Aurora Kinase B and Polo-like kinase 1

As shown previously (Fig. 3E–J. CDK4/6i-resistant cells progress through the cell cycle despite CDK4/6i treatment. Dysregulation of cell cycle regulatory genes may promote progression through G1 phase in resistant cells. Therefore, we explored the upregulation or downregulation of the cell cycle regulatory genes. The data are summarized in supplementary data 14. *CDKN2B* was upregulated in T47DrR, T47DrA, and T47DrP compared to that in T47D_controls. TFGβ was upregulated in T47DrR and T47DrP cells compared to that in T47D_controls. CDK4 and 6 upregulation was observed in T47DrA compared to that in T47D_controls. Polo-like kinase 1 (*PLK1*) upregulation was observed in MCF7rR, MCF7rA, and MCF7rP cells compared to that in MCF7 control cells. Aurora kinase B (*AukB*) was upregulated in MCF7rR and MCF7rP cells compared with that in MC7_controls. As AukB and PLK1 are targetable key cell cycle regulatory proteins, we tested the cytotoxicity of barasertib (AukB inhibitor) and volasertib (PLK1 inhibitor) in CDK4/6i-sensitive and-resistant MCF7 cells.

**Fig. 6:**
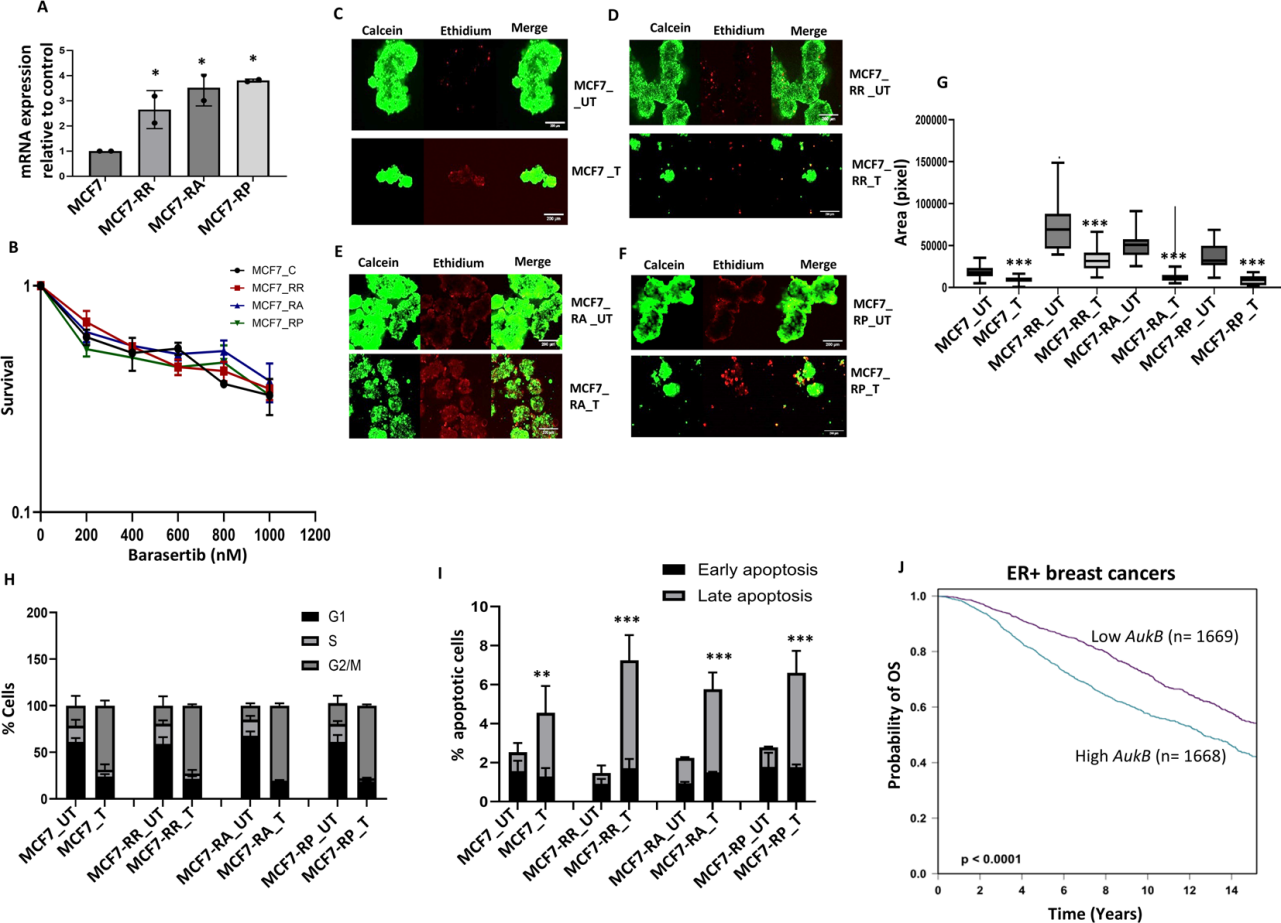
Barasertib sensitivity in CDK4/6i resistant MCF7 cells. (**A**) *AukB* transcript levels in MCF7 control and resistant cell lines. (**B**) Clonogenic assay of barasertib sensitivity in MCF7 control and resistant cell lines. (**C**) MCF7 control spheroids untreated (UT) or treated (T) with 800 nM of barasertib. (**D**) MCF7 ribociclib resistant spheroids untreated (UT) or treated (T) with 800 nM of barasertib. (**E**) MCF7 abemaciclib resistant spheroids untreated (UT) or treated (T) with 800 nM of barasertib. (**F**) MCF7 palbociclib resistant spheroids untreated (UT) or treated (T) with 800 nM of barasertib. (**G**) Spheroid size in MCF control and resistant cells untreated (UT) or treated (T) with 800 nM of barasertib. (**H**) Cell cycle progression in MCF control and resistant cells untreated (UT) or treated (T) with 800 nM of barasertib. (**I**) % apoptotic cells in MCF control and resistant cells untreated (UT) or treated (T) with 800 nM of barasertib. (**J**) AukB transcript expression and overall survival (OS) in clinical breast cancers. ‘*’- *p* ≤ 0.05, ‘**’- *p* ≤ 0.01, ‘***’- *p* ≤ 0.001. All figures are representative of 3 or more experiments. Error bars represent standard error of mean between experiments.

We confirmed *AukB* overexpression by qPCR in MCF7rR, MCF7rA, and MCF7rP cells compared to that in MCF7_control cells (Fig. 6A). Barasertib (AZD1152) is an orally bioavailable, small-molecule, dihydrogen phosphate prodrug of the potent pyrazoloquinazoline aurora kinase B inhibitor AZD1152-hydroxyquinazoline pyrazol anilide (HQPA). The blockade of AukB by AZD1152-HQPA disrupts spindle checkpoint functions and chromosome alignment, leading to the blockade of chromosome segregation and cytokinesis. AukB blockade results in the inhibition of cell division, cell proliferation, and induction of apoptosis, particularly in AukB-overexpressing cells^25^. Barasertib therapy increased cytotoxicity (Fig. 6B) and significantly reduced spheroid growth (Fig. 6C–G) in MCF7rR, MCF7rA, and MCF7rP spheroids, as well as in MCF7 control spheroids. The increased cytotoxicity was associated with profound G2/M cell cycle arrest (Fig. 6H) and significantly increased late apoptosis (Fig. 6I). We evaluated the clinicopathological significance of *AukB* transcripts in publicly available clinical breast cancer datasets (https://bcgenex.ico.unicancer.fr/BC-GEM/GEM-Accueil.php?js=1)^23,24^. High *AukB* mRNA expression was more common in Luminal B ER+ BCs than in Luminal A ER+ BCs (Supplementary Fig. 12A), high-risk Nottingham prognostic index (NPI) (Supplementary Fig. 12B), and was significantly associated with poor survival in the whole cohort (*p* < 0.0001) (Supplementary Fig. 12C), ER+ cohort (*p* < 0.0001) (Fig. 5J), but not in the ER- cohort (*p* = 0.79) (Supplementary Fig. 12D).

**Fig. 7:**
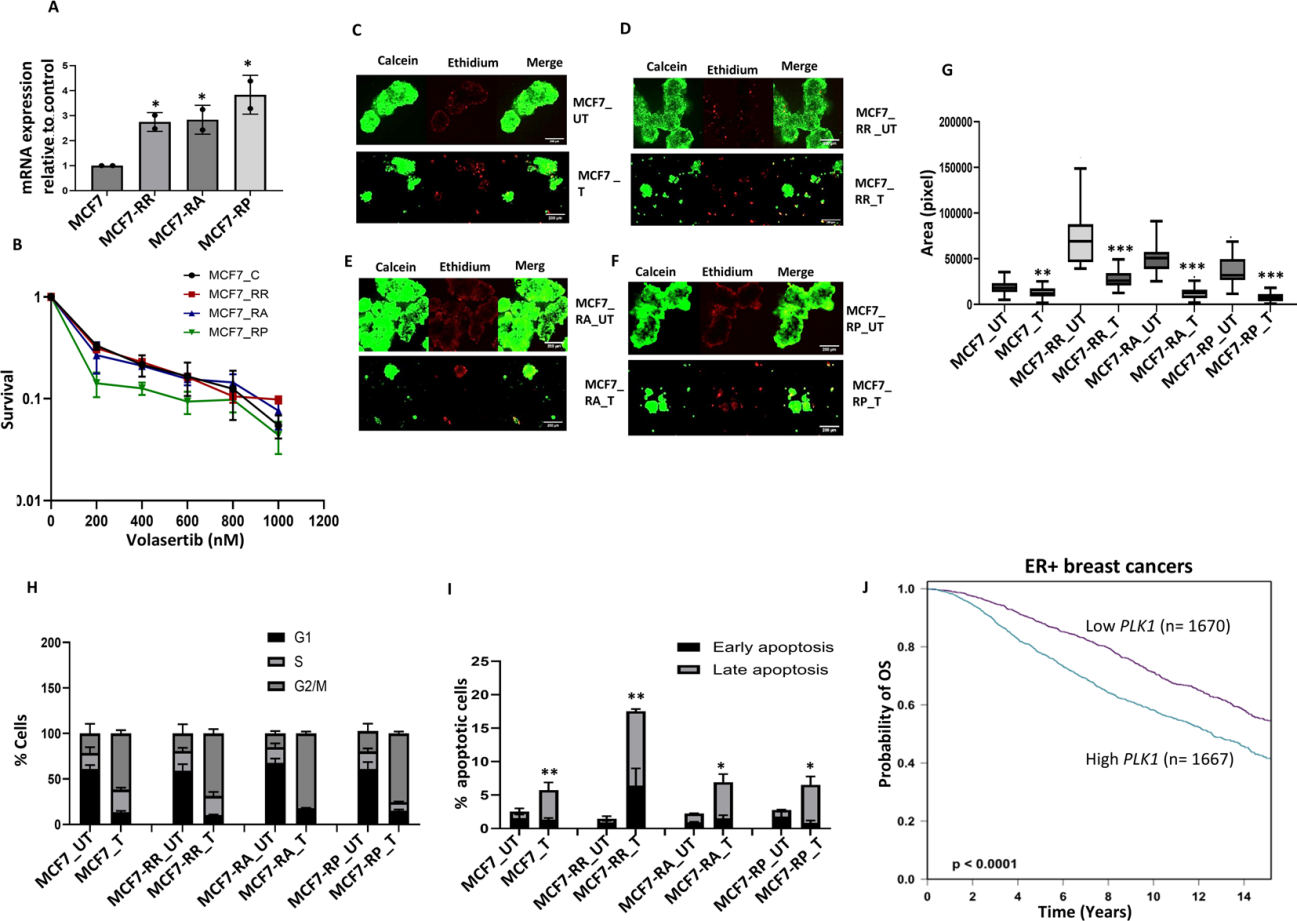
Volasertib sensitivity in CDK4/6i resistant MCF7 cells: (**A**) *PLK1* transcript levels in MCF7 control and resistant cell lines. (**B**) Clonogenic assay of volasertib sensitivity in MCF7 control and resistant cell lines. (**C**) MCF7 control spheroids untreated (UT) or treated (T) with 800 nM of volasertib. (**D**) MCF7 ribociclib resistant spheroids untreated (UT) or treated (T) with 800 nM of volasertib. (**E**) MCF7 abemaciclib resistant spheroids untreated (UT) or treated (T) with 800 nM of volasertib. (**F**) MCF7 palbociclib resistant spheroids untreated (UT) or treated (T) with 800 nM of volasertib. (**G**) Spheroid size in MCF control and resistant cells untreated (UT) or treated (T) with 800 nM of volasertib. (**H**) Cell cycle progression in MCF control and resistant cells untreated (UT) or treated (T) with 800 nM of volasertib. (**I**) % apoptotic cells in MCF control and resistant cells untreated (UT) or treated (T) with 800 nM of volasertib. (**J**) *PLK1* transcript expression and overall survival (OS) in clinical breast cancers. ‘*’- *p* ≤ 0.05, ‘**’- *p* ≤ 0.01, ‘***’- *p* ≤ 0.001. All figures are representative of 3 or more experiments. Error bars represent standard error of mean between experiments.

A previous study indicated that PLK1 inhibition in CCND1-driven breast cancer could be an anticancer strategy^26^. Moreover, the PLK1 Inhibitor Onvansertib, in combination with the PI3Kalpha Inhibitor Alpelisib, has been shown to overcome palbociclib resistance in PIK3CA-mutated ER breast cancer^27^. However, whether PLK1 blockade is effective in abemaciclib- or ribociclib-resistant ER+ breast cancer has not been described previously. In the current study, we observed *PLK1* upregulation not only in MCF7rP cells, but also in MCF7rR and MCF7rA cells. This upregulation was confirmed by qPCR (Fig. 7A). Volasertib (BI 6727) is an orally active, highly potent, and ATP-competitive Polo-like kinase 1 (PLK1) inhibitor with an IC_50_ of 0.87 nM. Volasertib inhibited PLK2 and PLK3 with IC_50_s of 5 nM and 56 nM, respectively. Volasertib induces mitotic arrest and apoptosis^28^. We investigated volasertib sensitivity in the control and CDK4/6i resistant cells. Volasertib therapy increased cytotoxicity (Fig. 7B) and reduced spheroid size in MCF7rR, MCF7rA, and MCF7rP spheroids as well as in MCF7 control spheroids (Fig. 7C–G). The increased cytotoxicity was associated with profound G2/M cell cycle arrest (Fig. 7H) and significantly increased apoptosis (Fig. 7I). In clinical breast cancer data sets (https://bcgenex.ico.unicancer.fr/BC-GEM/GEM-Accueil.php?js=1)^23,24^, *PLK1* mRNA overexpression was more common in Luminal B ER+ BCs than in Luminal A ER+ BCs (Supplementary Fig. 12E), high-risk Nottingham prognostic index (NPI) (Supplementary Fig. 12F), and significantly associated with poor survival in the whole cohort (*p* < 0.0001) (Supplementary Fig. 12G) and ER+ cohort (*p* < 0.0001) (Fig. 6J) but not in the ER- cohort (*p* = 0.25) (Supplementary Fig. 12H).

Taken together, these data provide evidence that G2/M blockade by clinical-grade AukB or PLK1 inhibitors may be a viable clinical strategy in ER+ breast cancer cells, including certain CDK4/6i-resistant cancers.

## Discussion

Ribociclib, palbociclib, and abemaciclib have revolutionized ER+ breast cancer therapeutics^4^. However, most patients eventually develop resistance and progress after several months of therapy. Therapeutic approaches beyond CDK4/6i are less well-defined and remain an area of unmet clinical need. Here, we induced acquired resistance to ribociclib, palbociclib, and abemaciclib, and investigated genomic alterations as well as therapeutic opportunities. We show that CDK4/6i-resistant BCs manifest aggressive phenotypes, bypass the G1/S phase of the cell cycle despite CDK4/6i treatment, and upregulate cell cycle genes promoting G2/M transition, including AukB and PLK1. While intrinsic resistance to CDK4/6i is less frequent (approximately 20% of breast cancers), acquired resistance is a common clinical challenge. Current clinical approaches beyond progression include a) change in endocrine therapy with PI3K or mTOR inhibitors or b) systemic chemotherapy in rapidly progressive disease. Emerging preclinical data suggest that multiple resistance mechanisms may be involved, including activation of cyclin D1, CDK4/6, CDK2, cyclin E1, E2F, PDK1, mTOR, CDK7, Wee1, PDLIM7, MDM2, FGFR1, KRAS, lysosomes, deactivation of Rb, FAT1, and CDH18^9^. Ultimately, these alterations lead to cell-cycle progression and promote cell survival. Preclinical data suggest that the blockade of CDK2, CDK7, FGFR1, and Wee1 is a promising approach in resistant cells^29^. In the current study, we showed that G2/M blockers such as barasertib (AZD1152, AukB inhibitor) or volasertib (BI 6727, a PLK1 inhibitor) are promising anti-cancer approaches in resistant cells.

While nuclear CDK4/6 is critical for the regulation of G1-S cell cycle progression, cytoplasmic roles, including migration, invasion, angiogenesis, and differentiation, have been described^3^. Additionally, CDK4 and CDK6 are involved in protein ubiquitination (thereby controlling protein stability), gene transcription, senescence, and cell metabolism^3^. CDK4/6i ribociclib, abemaciclib, and palbociclib, although specific CDK4/6 blockers, can induce differential transcriptional and proteomic responses, implying complex pharmacodynamic activity^12^. We hypothesized that the development of resistance to ribociclib, abemaciclib, or palbociclib will result in complex transcriptional changes that upregulate genes that ultimately promote aggressive phenotypes. As expected, CDK4/6i-resistant cells showed aggressive phenotypes, as evidenced by increased spheroid formation, invasion, proliferation, resistance to apoptosis, and cross-resistance to other CDK4/6i. Transcriptomics revealed distinct pathways upregulated in ribociclib, palbociclib, abemaciclib-resistant MCF7, and T47D cells. A key feature of resistant cells was that they continued cell cycling despite CDK4/6i therapy. Therefore, we explored the dysregulation of cell cycle regulatory genes and observed distinct patterns in the individual resistant cell lines. *CDKN2B* was upregulated in all resistant T47D cells compared to controls. In response to antimitogenic signals, monomeric CDK4 and CDK6 can be inhibited by the CDKN2 (INK4) family of proteins, including p15^INK4B^ (CDKN2B). A previous study showed that CDKN2A might prevent the binding of palbociclib to CDK4 and CDK6^30^. Overexpression of *CDKN2A* can not only reduce CDK4/6 activity but also induce acquired resistance by reducing the response to CDK4/6i^30^. Whether *CDKN2B* overexpression observed here may also induce resistance through a mechanism similar to that described by Green et al.^30^ will need to be confirmed in further mechanistic studies. Interestingly, *TGFβ* with known roles in proliferation^31^ was upregulated in T47DrR and T47DrP cells compared to MCF7 controls. Polo-like kinase 1 (*PLK1*) upregulation was observed in all resistant MCF7 cells compared to that in the MCF7 controls. PLK1 is a Ser/Thr kinase involved in cell cycle regulation primarily during G2/S and M phases^32^. Upregulation of *AukB*, a key regulator during the attachment of the mitotic spindle to the centromere^33^ was observed in MCF7rR and MCF7rP cells compared to MCF7 controls. We speculate that chronic CDK4/6 blockade could lead to rebound elevation of cyclins and other CDKs that are active in the G2/M phase of the cell cycle. For example, elevated WEE1 and CDK7 levels have been described in palbociclib-resistant cells^29^. As MCF7 cells are p53 wild-type and T47D cells are p53 mutant, it is likely that routes to resistance may be dependent on p53 status, which is critically involved in cell cycle regulation^34^. Interestingly, we observed an enrichment of p53 signalling in CDK4/6i-resistant cells, supporting this hypothesis. In addition, in a clinical cohort of 1005 tumors, we showed that *CDK6* overexpression in p53 mutated tumours was associated with poor survival while CDK6 overexpression did not show association with patient survival in p53 wild type tumours. Although our data were hypothesis generating, a previous preclinical study demonstrated that wild-type p53 is essential for the radiosensitizing ability of palbociclib in breast, lung, and colorectal cancer cell lines^35^. The efficacy of abemaciclib may also be dependent on p53 status in breast cancer^36^.

In ER+ clinical cohorts, we showed that overexpression of *PLK1* and *AukB* transcripts was significantly associated with an aggressive phenotype and poor survival. However, a limitation of the study is that the publicly available dataset does not allow multivariate analysis for prognostic evaluation. As PLK1 and AukB are potential anticancer drug targets, we tested clinical-grade small-molecule inhibitors of PLK1 and AukB in CDK4/6i-sensitive and-resistant cells.

In the current study, resistant cells remained sensitive to volasertib (a PLK1 inhibitor), which is associated with profound G2/M cell cycle arrest and increased apoptosis. A previous small study generated CDK4/6i-resistant, patient-derived organoids (PDO) from biopsies of patients who developed resistance to palbociclib. One PDO in this study showed sensitivity to a PLK1 inhibitor^37^. In another study, onvansertib (another PLK1 inhibitor), when combined with the PI3Kalpha inhibitor alpelisib, overcame palbociclib resistance in PIK3CA-mutated HR +  BCs^27^. In CCND1-driven breast cancer metastases with acquired palbociclib resistance, PLK1 inhibition showed antitumor activity in another preclinical study^26^. It is important to note that the above previous studies only investigated palbociclib-resistant models and did not include ribociclib- or abemaciclib-resistant ER+ BCs. Our data on ribociclib- and abemaciclib-resistant cells provide additional insight that PLK1 inhibitors could have broader applications irrespective of CDK4/6i resistance. Moreover, we also showed that ribociclib-, abemaciclib-, and palbociclib-resistant cells are also sensitive to barasertib (AukB inhibitor), which is associated with striking G2/M cell cycle arrest and increased apoptosis. Currently, barasertib and volasertib are undergoing clinical trials for haematological malignancies (www.clinicaltrials.gov). Whether they have a clinical impact on CDK4/6i-resistant ER+ BC remains to be established.

In conclusion, cell cycle upregulation leading to G2/M progression is a key route for CDK4/6i resistance. PLK1 or AukB inhibitors that block G2/M phase may have a clinical impact on CDK4/6i-resistant ER+ breast cancers.

## Electronic supplementary material

Below is the link to the electronic supplementary material.

Supplementary Material 1

Supplementary Material 2

Supplementary Material 3

Supplementary Material 4

Supplementary Material 5

Supplementary Material 6

Supplementary Material 7

Supplementary Material 8

Supplementary Material 9

Supplementary Material 10

Supplementary Material 11

Supplementary Material 12

Supplementary Material 13

Supplementary Material 14

Supplementary Material 15

Supplementary Material 16

Supplementary Material 17

Supplementary Material 18

Supplementary Material 19

Supplementary Material 20

Supplementary Material 21

Supplementary Material 22

Supplementary Material 23

Supplementary Material 24

Supplementary Material 25

Supplementary Material 26

Supplementary Material 27

Supplementary Material 28

Supplementary Material 29

Supplementary Material 30

Supplementary Material 31

Supplementary Material 32

Supplementary Material 33

Supplementary Material 34

Supplementary Material 35

Supplementary Material 36

Supplementary Material 37

Supplementary Material 38

Supplementary Material 39

## Data Availability

The data supporting this study can be found in the supplementary information file, and the corresponding author can make any material available upon request.

## References

[1] Kastan, M. B. & Bartek, J. Cell-cycle checkpoints and cancer. *Nature* **432**, 316–323 (2004). 15549093 10.1038/nature03097

[2] Baker, S. J. & Reddy, E. P. CDK4: A key player in the cell cycle, development, and cancer. *Genes Cancer* **3**, 658–669 (2012). 23634254 10.1177/1947601913478972 PMC3636745

[3] Fassl, A., Geng, Y. & Sicinski, P. CDK4 and CDK6 kinases: From basic science to cancer therapy. *Science* **375**, eabc1495 (2022). 35025636 10.1126/science.abc1495 PMC9048628

[4] Goel, S., Bergholz, J. S. & Zhao, J. J. Targeting CDK4 and CDK6 in cancer. *Nat. Rev. Cancer* **22**, 356–372 (2022). 35304604 10.1038/s41568-022-00456-3 PMC9149100

[5] Velasco-Velazquez, M. A. et al. Examining the role of cyclin D1 in breast cancer. *Future Oncol.* **7**, 753–765 (2011). 21675838 10.2217/fon.11.56

[6] Fuste, N. P. et al. Cytoplasmic cyclin D1 regulates cell invasion and metastasis through the phosphorylation of paxillin. *Nat. Commun.* **7**, 11581 (2016). 27181366 10.1038/ncomms11581 PMC4873647

[7] Kollmann, K. et al. A kinase-independent function of CDK6 links the cell cycle to tumor angiogenesis. *Cancer Cell* **30**, 359–360 (2016). 27505678 10.1016/j.ccell.2016.07.003 PMC5637299

[8] Uras, I. Z. et al. Palbociclib treatment of FLT3-ITD+ AML cells uncovers a kinase-dependent transcriptional regulation of FLT3 and PIM1 by CDK6. *Blood* **127**, 2890–2902 (2016). 27099147 10.1182/blood-2015-11-683581 PMC4920675

[9] Alvarez-Fernandez, M. & Malumbres, M. Mechanisms of sensitivity and resistance to CDK4/6 Inhibition. *Cancer Cell* **37**, 514–529 (2020). 32289274 10.1016/j.ccell.2020.03.010

[10] Watt, A. C. & Goel, S. Cellular mechanisms underlying response and resistance to CDK4/6 inhibitors in the treatment of hormone receptor-positive breast cancer. *Breast Cancer Res.* **24**, 17 (2022). 35248122 10.1186/s13058-022-01510-6 PMC8898415

[11] Adon, T., Shanmugarajan, D. & Kumar, H. Y. CDK4/6 inhibitors: A brief overview and prospective research directions. *RSC Adv.* **11**, 29227–29246 (2021). 35479560 10.1039/d1ra03820f PMC9040853

[12] Hafner, M. et al. Multiomics profiling establishes the polypharmacology of FDA-approved CDK4/6 inhibitors and the potential for differential clinical activity. *Cell Chem. Biol.* **26**, 1067–1080 (2019). 31178407 10.1016/j.chembiol.2019.05.005 PMC6936329

[13] McAndrew, N. P. & Finn, R. S. Clinical review on the management of hormone receptor-positive metastatic breast cancer. *JCO Oncol. Pract.* **18**, 319–327 (2022). 34637323 10.1200/OP.21.00384

[14] Finn, R. S. et al. Palbociclib and letrozole in advanced breast cancer. *N. Engl. J. Med.* **375**, 1925–1936 (2016). 27959613 10.1056/NEJMoa1607303

[15] Hortobagyi, G. N. et al. Updated results from MONALEESA-2, a phase III trial of first-line ribociclib plus letrozole versus placebo plus letrozole in hormone receptor-positive, HER2-negative advanced breast cancer. *Ann. Oncol.* **29**, 1541–1547 (2018). 29718092 10.1093/annonc/mdy155

[16] Goetz, M. P. et al. MONARCH 3: Abemaciclib as initial therapy for advanced breast cancer. *J. Clin. Oncol.* **35**, 3638–3646 (2017). 28968163 10.1200/JCO.2017.75.6155

[17] Johnston, S. et al. MONARCH 3 final PFS: A randomized study of abemaciclib as initial therapy for advanced breast cancer. *NPJ Breast Cancer* **5**, 5 (2019). 30675515 10.1038/s41523-018-0097-z PMC6336880

[18] Hortobagyi, G. N. et al. Overall survival with ribociclib plus letrozole in advanced breast cancer. *N. Engl. J. Med.* **386**, 942–950 (2022). 35263519 10.1056/NEJMoa2114663

[19] Goetz, M. P. et al. Abemaciclib plus a nonsteroidal aromatase inhibitor as initial therapy for HR+ , HER2- advanced breast cancer: Final overall survival results of MONARCH 3. *Ann. Oncol.* **35**, 718–727 (2024). 38729566 10.1016/j.annonc.2024.04.013

[20] Slamon, D. J. et al. Overall survival with palbociclib plus letrozole in advanced breast cancer. *J. Clin. Oncol.* **42**, 994–1000 (2024). 38252901 10.1200/JCO.23.00137 PMC10950136

[21] McShane, L. M. et al. REporting recommendations for tumour MARKer prognostic studies (REMARK). *Br. J. Cancer* **93**, 387–391 (2005). 16106245 10.1038/sj.bjc.6602678 PMC2361579

[22] Camp, R. L., Dolled-Filhart, M. & Rimm, D. L. X-tile: A new bio-informatics tool for biomarker assessment and outcome-based cut-point optimization. *Clin. Cancer Res.* **10**, 7252–7259 (2004). 15534099 10.1158/1078-0432.CCR-04-0713

[23] Jezequel, P. et al. bc-GenExMiner: an easy-to-use online platform for gene prognostic analyses in breast cancer. *Breast Cancer Res. Treat.* **131**, 765–775 (2012). 21452023 10.1007/s10549-011-1457-7

[24] Jézéquel, P. et al. bc-GenExMiner 4.5: New mining module computes breast cancer differential gene expression analyses. *Database* **2021**, baab007 (2021). 33599248 10.1093/database/baab007 PMC7904047

[25] Mortlock, A. A. et al. Discovery, synthesis, and in vivo activity of a new class of pyrazoloquinazolines as selective inhibitors of aurora B kinase. *J. Med. Chem.* **50**, 2213–2224 (2007). 17373783 10.1021/jm061335f

[26] Montaudon, E. et al. PLK1 inhibition exhibits strong anti-tumoral activity in CCND1-driven breast cancer metastases with acquired palbociclib resistance. *Nat. Commun.* **11**, 4053 (2020). 32792481 10.1038/s41467-020-17697-1 PMC7426966

[27] Sreekumar, S. et al. Combination of the PLK1 inhibitor onvansertib and the PI3Kalpha inhibitor alpelisib overcomes palbociclib resistance in PIK3CA-mutated HR+ breast cancer. *CanRes* **84**(9 Supplement), Abstract PO2-04-09 (2024).

[28] Gjertsen, B. T. & Schoffski, P. Discovery and development of the Polo-like kinase inhibitor volasertib in cancer therapy. *Leukemia* **29**, 11–19 (2015). 25027517 10.1038/leu.2014.222 PMC4335352

[29] Scheidemann, E. R. & Shajahan-Haq, A. N. Resistance to CDK4/6 inhibitors in estrogen receptor-positive breast cancer. *Int. J. Mol. Sci.* **22**, (2021). 10.3390/ijms222212292 PMC8625090 34830174

[30] Green, J. L. et al. Direct CDKN2 modulation of CDK4 alters target engagement of CDK4 inhibitor drugs. *Mol. Cancer Ther.* **18**, 771–779 (2019). 30837298 10.1158/1535-7163.MCT-18-0755

[31] Zhang, Y., Alexander, P. B. & Wang, X. F. TGF-beta family signaling in the control of cell proliferation and survival. *Cold Spring Harb. Perspect. Biol.* **9**, (2017). 10.1101/cshperspect.a022145 PMC5378054 27920038

[32] Iliaki, S., Beyaert, R. & Afonina, I. S. Polo-like kinase 1 (PLK1) signaling in cancer and beyond. *Biochem. Pharmacol.* **193**, 114747 (2021). 34454931 10.1016/j.bcp.2021.114747

[33] Borah, N. A. & Reddy, M. M. Aurora Kinase B inhibition: A potential therapeutic strategy for cancer. *Molecules* **26**, (2021). 10.3390/molecules26071981 PMC8037052 33915740

[34] Chen, J. The cell-cycle arrest and apoptotic functions of p53 in tumor initiation and progression. *Cold Spring Harb. Perspect. Med.* **6**, a026104 (2016). 26931810 10.1101/cshperspect.a026104 PMC4772082

[35] Fernandez-Aroca, D. M. et al. P53 pathway is a major determinant in the radiosensitizing effect of Palbociclib: Implication in cancer therapy. *Cancer Lett.* **451**, 23–33 (2019). 30872077 10.1016/j.canlet.2019.02.049

[36] Wang, B. et al. Pharmacological CDK4/6 inhibition reveals a p53-dependent senescent state with restricted toxicity. *EMBO J.* **41**, e108946 (2022). 34985783 10.15252/embj.2021108946 PMC8922251

[37] Hanker, A. B. et al. A platform of CDK4/6 inhibitor-resistant patient-derived breast cancer organoids illuminates mechanisms of resistance and therapeutic vulnerabilities. *CanRes.* **82**(4 SUPPL), Abstract PD2-01 (2022).

