# Supplementary material for: Investigation of ribociclib, abemaciclib and palbociclib resistance in ER+ breast cancer cells reveal potential therapeutic opportunities
Source: Sci Rep. 2025 Aug 5;15:28579. doi: 10.1038/s41598-025-11052-4 (PMC12325956; doi:10.1038/s41598-025-11052-4)

SUPPLEMENTARY MATERIAL AND METHODS

Immunohistochemical evaluation

Study cohort

CDK4 and CDK6 protein expressions were evaluated on a well-characterised primary BC series (n = 1055) from patients presented at Nottingham City Hospital, NHS Trust, Nottingham, United Kingdom. Clinical and tumour characteristics including patient's age at diagnosis, histological tumour type, grade, tumour size, lymph node status, Nottingham Prognostic Index (NPI), and lympho-vascular invasion (LVI), were available (**Supplementary Table 2**). In addition, the outcome data in the form of BC specific survival (BCSS), defined as time (in months) from the date of primary surgical treatment to the time of death by BC, and distant metastasis-free survival (DMFS) defined as the time (in months) from the surgery until the first event of distant metastasis were also collected from patients` records. Adjuvant treatment was given according to the institutional protocols. Information regarding oestrogen receptor (ER), progesterone receptor (PR), and human epidermal growth factor 2 (HER2) (23-25) were available. Tumours were also classified based on ER, PR and HER2 into three molecular subtypes (luminal, triple-negative (TN), HER2-enriched) as following: ER+ HER2- (luminal BC), ER-, PR- and HER2- (TNBC) while tumours with HER2+ (HER2 enriched) (26). In view of the importance of p53 and it is interaction with CDK4 and CDK6 (27), the data regarding immunohistochemical expression of p53 were available from a previous study and used for comparative analysis with CDK4 and CDK6. For the importance of Ki67 as surrogate of cellular proliferation in BC and its role in cell cycle, Ki67 levels as measured in previous study were used in comparative analysis with CDK4 and CDK6 (DOI: 10.1111/his.14781).

CDK4, CDK6 and p53 protein expression evaluation

Prior to immunohistochemistry (IHC) staining of the tissue sections, the specificity of Anti-CDK4 monoclonal antibody (DCS-31), Invitrogen, UK) and Anti CDK6 monoclonal antibody (SD20-50), Invitrogen, UK were validated by western blotting using cell lysates of MCF7, SKBR3, HeLa and MCF10 obtained from American Type Culture Collection, Rockville, MD, USA. The CDK4 primary antibody was used at 1:200 dilution while CDK6 was used at 1.1000 dilution. Proteins were detected using IRDye 800CW fluorescent secondary antibodies (1:5 000 dilution, LI-COR Biosciences) and the Odyssey Fc with Image Studio 4.0 (LI-COR Biosciences) was used to visualise the bands. Anti-β-actin primary antibody (Sigma-Aldrich) was used as a loading control (1: 5000). Tumour samples were arrayed using the Grand Master® (3D HISTECH®, Budapest, Hungary) as described previously (28). Tissue sections using the Novocastra Novolink™ Polymer Detection Systems kit (Code: RE7280-K, Leica, Biosystems, Newcastle, UK) of 4 μm thick were dewaxed and endogenous peroxidase activity was blocked with 0.3% hydrogen peroxide in methanol for 10 min. Antigen retrieval was performed in citrate buffer pH 6.0 using a microwave (Whirlpool JT359 Jet Chef 1000 W) for 20 min. Mouse monoclonal CDK4 and CDK6 were diluted at 1:20 and 1:15, respectively in Leica antibody diluent (RE AR9352, Leica, Biosystems, UK) and incubated for 60 min at room temperature. Normal kidney tissue was used as a positive control for CDK4 while normal liver tissue was used as a positive control for CDK6.Negative control was obtained by omitting the primary antibodies. The sections were counterstained with haematoxylin.

CDK4 and CDK6 antibodies showed both nuclear and cytoplasmic expression. Therefore, the percentage of positive tumour cells were calculated for both sub localisation. A semi-quantitative evaluation was used to assess a modified

histochemical score (H-score) of both markers and the final H-score was obtained by giving a range of 0 to 300 as previously described (29, 30). X-tile bioinformatics software version 3.6.1 (School of Medicine, Yale University, New Haven, CT, USA) was used (31) to categorise CDK4 and CDK6 H-scores into low and high expression. H-score of 110 and 50 were considered the best cut-offs of nuclear and cytoplasmic expressions of CDK4, respectively. The optimal cut points for both nuclear and cytoplasmic expressions of CDK6 were 80 and 40, respectively. CDK4 and CDK6 expressions were compared with the available clinicopathological parameters and with the outcome data. Immunostaining for p53 showed nuclear expression and its score was evaluated using a semi-quantitative system. H- score from 0-300 was calculated for each case. 10% cut-off was used as the optimal cut-off of categorisation of p53 expression into negative (wild type) and positive (mutant) tumours. For the interaction between p53, CDK4 and CDK6 markers, the whole cohort was categorised according to p53 status into wild type tumours (p53-) and p53 mutant tumours (p53+) and clinicopathological variables were investigated.

Statistical analysis

Statistical Package for the Social Sciences software v.27.0 (SPSS, Chicago, IL, USA) was used for statistical analysis. Chi-square test was used for analysis of categorical data. Outcome analysis was assessed using Kaplan–Meier curves and the log-rank test. The association of CDK4 and CDK6 expressions with the different molecular classes of breast cancer was also evaluated. Cox regression models was used for the multivariate analysis. For statistical analysis, Ki67 expression levels were categorised into low and high proliferative tumours based on 14% cut-off (DOI: 10.1093/jnci/djp082). Estimated hazard ratio (HR) and their 95% confidence interval

(95% CI) were calculated. For all tests, $P < 0.05$ (two-tailed) were statistically significant.

This study was approved by the Yorkshire & the Humber - Leeds East Research Ethics Committee (REC Reference: 19/YH/0293) under the IRAS Project ID: 266925. Data collected were fully anonymised.

Transcriptomic analyses: Prognostic significance of CDK4 *mRNA*, CDK6 *mRNA* was evaluated in a cohort of 5031 breast cancers where gene expression dataset was publicly available at bc-GenExMiner (http://bcgenex.ico.unicancer.fr/BC-GEM/GEM-Accueil.php?js=1) for the analysis.

RNA sequencing and bioinformatics

RNA sequencing and bioinformatics service was provided by Novogene Europe (novogene-europe.com).

Sample Quality Control

The Sequenced Reads/raw reads often contain low quality reads or reads with adapters, which will affect the analysis quality and reliability. To avoid this, it's necessary to filter the raw reads and get the clean reads. The procedure of raw reads filtering is as follows:

(1) Remove reads containing adapters;

(2) Remove reads containing N > 10% (N represents base that could not be determined);

(3) Remove low quality reads: The Qscore (Quality value) of over 50% bases of the read is <= 5.

Library Construction, Quality Control and Sequencing

Messenger RNA was purified from total RNA using poly-T oligo-attached magnetic beads. After fragmentation, the first strand cDNA was synthesized using random hexamer primers, followed by the second strand cDNA synthesis using either dUTP for directional library or dTTP for non-directional library. For the non-directional library, it was ready after end repair, A-tailing, adapter ligation, size selection, amplification, and purification. For the directional library, it was ready after end repair, A-tailing, adapter ligation, size selection, USER enzyme digestion, amplification, and

Purification The library was checked with Qubit and real-time PCR for quantification

and bioanalyzer for size distribution detection. Quantified libraries will be pooled and sequenced on Illumina platforms, according to effective library concentration and data amount.

Bioinformatics Analysis Pipeline

Data Quality Control

Raw data (raw reads) of fastq format were firstly processed through in-house perl scripts. In this step, clean data (clean reads) were obtained by removing reads containing adapter, reads containing ploy-N and low quality reads from raw data. At the same time, Q20, Q30 and GC content the clean data were calculated. All the downstream analyses were based on the clean data with high quality.

Reads mapping to the reference genome

Reference genome and gene model annotation files were downloaded from genome website directly. Index of the reference genome was built using Hisat2 v2.0.5 and paired-end clean 1 reads were aligned to the reference genome using Hisat2 v2.0.5. We selected Hisat2 as the mapping tool for that Hisat2 can generate a database of splice junctions based on the gene model annotation file and thus a better mapping result than other non-splice mapping tools.

Quantification of gene expression level

featureCounts v1.5.0-p3 was used to count the reads numbers mapped to each gene. And then FPKM of each gene was calculated based on the length of the gene and reads count mapped to this gene. FPKM, expected number of Fragments Per Kilobase of transcript sequence per Millions base pairs sequenced, considers the effect of sequencing depth and gene length for the reads count at the same time, and is currently the most commonly used method for estimating gene expression levels.

Principal Component Analysis

Principal component analysis (PCA) is commonly used to evaluate intergroup differences and intragroup sample duplication. PCA uses the linear algebra calculation method to reduce dimension and extract principal components from tens of thousands of gene variables. We performed PCA analysis on the gene expression value (FPKM) of all samples, as shown in the figure below. Under ideal conditions, the samples between groups should be dispersed and the samples within groups should be gathered together.

Cluster Analysis

All the differentially expressed genes in the comparison group were pooled as the differential gene set. For more than two groups of experiments, cluster analysis [cluster Profiler software (version 3.10.1) (https://bioconductor.org/packages/release/bioc/html/clusterProfiler.html)] can be carried out on different gene sets and genes with similar expression patterns can be clustered together. We used the mainstream hierarchical clustering to cluster the fpkm values of genes, and homogenized the row (Z-score). The genes or samples with similar expression patterns in the heat map will be gathered together. The color in each grid reflects not the gene expression value, but the value obtained after homogenizing the expression data rows (generally between - 2 and 2). Therefore, the colors in the heat map can only be compared horizontally (the expression of the same gene in different samples), but not vertically (the same sample). There are not only inter group clustering, but also inter sample clustering. The final report shows the clustering among samples.

Differential expression analysis

(For DESeq2 with biological replicates) Differential expression analysis of two conditions/groups (two biological replicates per condition) was performed using the DESeq2Rpackage (1.20.0). DESeq2 provide statistical routines for determining differential expression in digital gene expression data using a model based on the negative binomial distribution. The resulting P-values were adjusted using the Benjamini and Hochberg's approach for controlling the false discovery rate. Genes with an adjusted P-value <=0.05found by DESeq2 were assigned as differentially expressed. (For edgeR without biological replicates) Prior to differential gene expression analysis, for each sequenced library, the read counts were adjusted by edgeR program package through one scaling normalized factor. Differential expression analysis of two conditions was performed using the edgeR package (3.22.5). The P values were adjusted using the Benjamini & Hochberg method. Corrected P-value of 0.05 and absolute foldchange of 2 were set as the threshold for significantly differential expression.

Enrichment analysis of differentially expressed genes

Gene Ontology (GO) enrichment analysis of differentially expressed genes was implemented by the cluster Profiler R package (https://bioconductor.org/packages/release/bioc/html/clusterProfiler.html), in which gene length bias was corrected. GO terms with corrected P value less than 0.05 were considered significantly enriched by differential expressed genes. KEGG is a database resource for understanding high-level functions and utilities of the biological system, such as the cell, the organism and the ecosystem, from molecular-level information, especially large-scale molecular datasets generated by genome sequencing and other high-through put experimental technologies (http://www.genome.jp/kegg/). We used clusterProfiler R package (version 3.10.1)

(https://bioconductor.org/packages/devel/bioc/html/clusterProfiler.html) to test the statistical enrichment of differential expression genes in KEGG pathways [1,2, 3]. The Reactome database brings together the various reactions and biological pathways of human model species. Reactome pathways with corrected P value less than 0.05 were considered significantly enriched by differential expressed genes. The DO (Disease Ontology) database describes the function of human genes and diseases. DO pathways with corrected P value less than 0.05 were considered significantly enriched by differential expressed genes. The DisGeNET database integrates human disease-related genes. DisGeNET pathways with corrected P value less than 0.05 were considered significantly enriched by differential expressed genes. We used cluster Profiler software (version 3.10.1) (https://bioconductor.org/packages/release/bioc/html/clusterProfiler.html) to test the statistical enrichment of differentially expressed genes in the Reactome pathway, the DO pathway, and the DisGeNET pathway.

Gene Set Enrichment Analysis

Gene Set Enrichment Analysis (GSEA) is a computational approach to determine if a pre- defined Gene Set can show a significant consistent difference between two biological states. The genes were ranked according to the degree of differential expression in the two samples, and then the predefined Gene Set were tested to see if they were enriched at the top or bottom of the list. Gene set enrichment analysis can include subtle expression changes. We use the local version of the GSEA analysis tool http://www.broadinstitute.org/gsea/index.jsp as part of the Molecular Signatures Database (MSigDB), using the predefined gene sets GO、KEGG、Reactome、DO and DisGeNET for GSEA independently (reference:

https://pubmed.ncbi.nlm.nih.gov/16199517/) (reference: https://pubmed.ncbi.nlm.nih.gov/21546393/).

Supplementary References

1. Kanehisa, M., Furumichi, M., Sato, Y., Matsuura, Y. and Ishiguro-Watanabe, M.; KEGG: biological systems database as a model of the real world. Nucleic Acids Res. 53, D672-D677 (2025).

2. Kanehisa, M; Toward understanding the origin and evolution of cellular organisms. Protein Sci. 28, 1947-1951 (2019)

3. Kanehisa, M. and Goto, S.; KEGG: Kyoto Encyclopedia of Genes and Genomes. Nucleic Acids Res. 28, 27-30 (2000).

Supplementary Table 1: Percentage of cells in various stages of cell cycle.

Cell line	G1 (Mean ± SD*)	S phase (Mean ± SD*)	G2/M (Mean ± SD*)
**T47D_C**	53±1	26±6	21±6.2
**T47D_C_R**	72.3± 4.1	5.3±1.5	22.3±2.8
**T47D_RR**	56±1.7	18±3.2	25.3±4.5
**T47D_RR_R**	65.6±0.5	10.3±2	24±1.7
**T47D_C_A**	76.5±4.9	11±1.4	21±3.5
**T47D_RA**	48±1	18.3±3.5	12.5±3.2
**T47D_RA_A**	59±1.2	23±1	18±1.4
**T47D_C_P**	86.5±3.5	1.5±0.7	21±6.2
**T47D_RP**	49±1	18±	12±2.8
**T47D_RP_P**	63.5±3.5	18.5±0.7	33±1
**MCF_C**	53±1.4	30±4.8	18±2.8
**MCF7_C_R**	72.75±3.7	17.25±4.7	7.5±0.57
**MCF7_RR**	45±2.1	30.75±4.11	24.25±2.06
**MCF7_RR_R**	54±1.4	19.25±0.95	26.75±2.21
**MCF7_C_A**	85.25±6.1	6.7±4.03	8±2
**MCF7_RA**	40.25±0.5	37.25±4.03	22.5±4.4
**MCF7_RA_A**	60.75±1.7	12.5±1.9	26±1.7
**MCF7_C_P**	84.25±1.5	7±1.7	8±0.8
**MCF7_RP**	46.5±1.2	31.6±0.9	22.5±0.9
**MCF7_RP_P**	63.7±2.9	11.6±1.2	24.75±2.2

*SD: Standard deviation

Supplementary Table 2. Clinicopathological characteristics of the study cohort

Variables	No (%)
Age at diagnosis	
<50	327 (31%)
≥50	728 (69%)
Menopausal status	
Premenopausal	363 (34%)
Post-menopausal	692 (66%)
Tumour size	
≤ 2cm	634 (60%)
> 2cm	421 (40%)
Grade	
Grade 1	152 (15%)
Grade 2	424 (40%)
Grade 3	479 (45%)
Tubule formation	
1	73 (7%)
2	305 (29%)
3	677 (64%)
Polymorphism	
1	17 (2%)
2	295 (28%)
3	743 (70%)
Mitosis	
1	502 (48%)
2	212 (20%)
3	341 (32%)
Histologic tumour types	
No Special Type (NST)	689 (65%)
Lobular	90 (9%)
Other special types	43 (4%)
NST mixed	233 (22%)
Lymhovascular invasion	
Absent	759 (72%)
Present	296 (28%)
Lymph node status	
Absent	647 (61%)
Present	408 (39%)
Nottingham prognostic index	
Good prognostic group	344 (33%)
Moderate prognostic group	538 (51%)
Poor prognostic group	173 (16%)
Ki67 expression	
Low ≤ 14%	417 (53%)
High >14%	374 (47%)
Molecular subtypes	
Luminal A	383 (42%)
Luminal B	337 (37%)
HER2 enriched	52 (6%)
Triple negative	149 (15%)

Ki67 was assessed on 791 samples

Supplementary Table 3. Nuclear and cytoplasmic CDK4 co-expression and clinicopathological parameters in p53 wild type tumours.

**Categories**	N-/C- n (%)	N+/C+ n (%)	N+/C- n (%)	N-/C+ n (%)	**X^2** ***P*-value**
**Age at diagnosis (years)**					6.133
<50	41 (22)	41 (22)	68 (35)	40 (21)	0.105
≥50	74 (17)	92 (22)	194 (45)	68 (16)	
**Menopausal status**					3.598
Premenopausal	43 (20)	46 (22)	80 (38)	43 (20)	0.308
Post-menopausal	72 (18)	87 (21)	182 (45)	65 (16)	
**Tumour size**					0.729
≤ 2cm	69 (19)	76 (20)	161 (43)	66 (18)	0.866
> 2cm	46 (19)	57 (23)	101 (41)	42 (17)	
**Tumour grade**					17.470
Grade 1	19 (18)	27 (26)	49 (46)	11 (10)	**0.008**
Grade 2	53 (18)	55 (18)	141 (48)	46 (16)	
Grade 3	43 (19)	51 (24)	72 (33)	51 (24)	
**Histologic types**					52.779
No special type (NST)	73 (20)	87 (23)	125 (34)	87 (23)	**<0.0001**
Lobular	13 (20)	4 (6)	46 (71)	2 (3)	
Other special types	6 (21)	8 (29)	11 (39)	3 (11)	
Mixed NST	23 (15)	34 (22)	80 (52)	16 (11)	
**Molecular subtype**					17.864
Luminal A	43 (17)	55 (21)	124 (48)	34 (13)	**0.037**
Luminal B	2 (18)	1 (9)	6 (55)	2 (18)	
Triple negative	17 (29)	11 (19)	16 (28)	14 (24)	

HER2 enriched	38 (18)	55 (27)	76 (37)	37 (18)	
**Lymph node status**					1.830
Negative	74 (19)	76 (20)	166 (44)	66 (17)	0.608
Positive	41 (17)	57 (24)	96 (41)	42 (18)	
**Lymphovascular invasion**					6.970
Absent	84 (18)	90 (20)	207 (45)	76 (17)	0.073
Present	31 (19)	43 (27)	55 (34)	32 (20)	
**Nottingham Prognostic index**					7.682<br>0.262
Good prognostic group	45 (20)	43 (19)	109 (48)	31 (13)	
Moderate prognostic group	57 (19)	72 (23)	118 (38)	60 (20)	
Poor prognostic group	13 (16)	18 (22)	35 (42)	17 (20)	
**Ki67 expression**					3.398
Low ≤14	48 (18)	58 (22)	124 (46)	37 (14)	0.334
High >14	35 (17)	44 (22)	82 (41)	40 (20)	

HER2, human epidermal growth factor receptor 2 Significant P values are in bold

Supplementary Table 4. Nuclear and cytoplasmic CDK4 co-expression and clinicopathological parameters in p53 mutated tumours

Categories	N-/C-<br>n (%)	N+/C+<br>n (%)	N+/C-<br>n (%)	N-/C+<br>n (%)	**X^2**<br>***P*-value**
**Age at diagnosis (years)**					6.041
<50	16 (14)	23 (20)	44 (38)	32 (28)	0.110
≥50	43 (22)	40 (20)	82 (41)	35 (17)	
**Menopausal status**					6.049
Premenopausal	17 (14)	23 (20)	45 (38)	33 (28)	0.109
Post-menopausal	42 (22)	40 (20)	81 (41)	34 (17)	
**Tumour size**					1.550
≤ 2cm	35 (19)	34 (18)	76 (42)	35 (19)	0.671
> 2cm	24 (18)	29 (21)	50 (37)	32 (24)	
**Tumour grade**					12.570
Grade 1	4 (14)	8 (27)	15 (52)	2 (7)	0.050
Grade 2	15 (20)	11 (15)	37 (50)	11 (15)	
Grade 3	40 (19)	44 (21)	74 (35)	54 (25)	
**Histologic types**					22.109
No special type (NST)	50 (20)	56 (22)	87 (34)	61 (24)	**0.009**
Lobular	1 (11)	1 (11)	6 (67)	1 (11)	
Other special types	2 (29)	2 (29)	3 (42)	0 (0)	
Mixed NST	6 (13)	4 (9)	30 (67)	5 (11)	
**Molecular subtype**					11.125
Luminal A	11 (18)	14 (22)	31 (49)	7 (11)	0.267
Luminal B	8 (28)	4 (13)	9 (31)	8 (28)	
Triple negative	18 (24)	14 (18)	24 (31)	21 (27)	

HER2 enriched	19 (17)	23 (21)	42 (38)	27 (24)	
**Lymph node status**					3.773
Negative	40 (21)	34 (18)	72 (38)	44 (23)	0.287
Positive	19 (15)	29 (23)	54 (43)	23 (19)	
**Lymphovascular invasion**					6.152
Absent	45 (22)	37 (18)	78 (37)	48 (23)	0.104
Present	14 (13)	26 (24)	48 (45)	19 (18)	
**Nottingham Prognostic Index**					8.228<br>0.222
Good prognostic group	13 (18)	15 (21)	34 (48)	9 (13)	
Moderate prognostic group	36 (21)	30 (17)	65 (37)	44 (25)	
Poor prognostic group	10 (15)	18 (26)	27 (39)	14 (20)	
**Ki67 expression**					3.462
Low ≤14	15 (18)	19 (24)	36 (44)	11 (14)	0.326
High >14	31 (22)	26 (18)	53 (38)	31 (22)	

HER2, human epidermal growth factor receptor 2 Significant P values are in **bold**

Supplementary Table 5. Nuclear and cytoplasmic CDK6 co-expression and clinicopathological parameters in p53 wild type tumours

**Categories**	N-/C- n (%)	N+/C+ n (%)	N+/C- n (%)	N-/C+ n (%)	**X^2** ***P*-value**
**Age at diagnosis (years)**					14.493
<50	98 (53)	4 (2)	37 (20)	47 (25)	**0.002**
≥50	178 (43)	2 (1)	139 (33)	100 (24)	
**Menopausal status**					8.581
Premenopausal	101 (49)	4 (2)	47 (23)	55 (26)	**0.035**
Post-menopausal	175 (44)	2 (1)	129 (32)	92 (23)	
**Tumour size**					4.862
≤ 2cm	161 (44)	5 (1)	115 (32)	82 (23)	0.182
> 2cm	115 (47)	1 (1)	61 (25)	65 (27)	
**Tumour grade**					46.884
Grade 1	30 (29)	3 (3)	50 (49)	19 (19)	**<0.0001**
Grade 2	125 (44)	1 (1)	91 (32)	64 (23)	
Grade 3	121 (54)	2 (1)	35 (16)	64 (29)	
**Histologic types**					61.943
No special type (NST)	194 (53)	2 (1)	69 (19)	98 (27)	**0.0001**
Lobular	26 (42)	1 (2)	26 (42)	9 (14)	
Other special types	10 (36)	2 (7)	12 (43)	4 (14)	
Mixed NST	46 (30)	1 (1)	69 (45)	36 (24)	
**Molecular subtype**					20.164
Luminal A	26 (37)	2 (3)	25 (36)	17 (24)	**0.017**
Luminal B	17 (53)	0 (0)	7 (22)	8 (25)	
Triple negative	48 (61)	0 (0)	9 (12)	21 (27)	

HER2 enriched	66 (60)	1 (1)	22 (20)	21 (19)	
**Lymph node status**					0.236
Negative	100 (53)	2 (1)	44 (24)	42 (22)	0.972
Positive	71 (55)	1 (1)	28 (21)	30 (23)	
**Lymphovascular invasion**					2.828
Absent	116 (55)	1 (1)	44 (21)	50 (23)	0.419
Present	55 (51)	2 (2)	28 (26)	22 (21)	
**Nottingham Prognostic index**					13.267 **0.039**
Good prognostic group	30 (44)	2 (3)	22 (32)	14 (21)	
Moderate prognostic group	99 (55)	1 (1)	41 (23)	37 (21)	
Poor prognostic group	42 (58)	0 (0)	9 (13)	21 (29)	
**Ki67 expression**					8.927
Low ≤14	34 (40)	2 (2)	27 (32)	22 (26)	**0.030**
High >14	86 (59)	1 (1)	28 (19)	31 (21)	

HER2, human epidermal growth factor receptor 2   Significant P values are in **bold**

Supplementary Table 6. Nuclear and cytoplasmic CDK6 co-expression and clinicopathological parameters in p53 mutated tumours

Categories	N-/C- n (%)	N+/C+ n (%)	N+/C- n (%)	N-/C+ n (%)	**X^2** ***P*-value**
**Age at diagnosis (years)**					7.329
<50	70 (61)	0 (0)	18 (16)	27 (23)	0.062
≥50	101 (50)	3 (1)	54 (27)	45 (22)	
**Menopausal status**					6.339
Premenopausal	71 (59)	0 (0)	20 (16)	30 (25)	0.096
Post-menopausal	100 (51)	3 (2)	52 (26)	42 (21)	
**Tumour size**					7.941
≤ 2cm	98 (54)	3 (2)	47 (26)	33 (18)	**0.047**
> 2cm	73 (53)	0 (0)	25 (18)	39 (29)	
**Tumour grade**					17.222
Grade 1	9 (35)	1 (4)	10 (38)	6 (23)	**0.008**
Grade 2	37 (50)	1 (1)	25 (33)	12 (16)	
Grade 3	125 (57)	1 (1)	37 (17)	54 (25)	
**Histologic types**					32.747
No special type (NST)	154 (59)	1 (1)	50 (19)	56 (21)	**0.0001**
Lobular	2 (26)	0 (0)	3 (38)	3 (38)	
Other special types	3 (43)	1 (14)	2 (29)	1 (14)	
Mixed NST	12 (29)	1 (2)	17 (40)	12 (29)	
**Molecular subtype**					20.164
Luminal A	26 (37)	2 (3)	25 (36)	17 (24)	**0.017**
Luminal B	17 (53)	0 (0)	7 (22)	8 (25)	
Triple negative	48 (61)	0 (0)	9 (12)	21 (27)	

HER2 enriched	66 (60)	1 (1)	22 (20)	21 (19)	
**Lymph node status**					0.236
Negative	100 (53)	2 (1)	44 (24)	42 (22)	0.972
Positive	71 (55)	1 (1)	28 (21)	30 (23)	
**Lymphovascular invasion**					2.828
Absent	116 (55)	1 (1)	44 (21)	50 (23)	0.419
Present	55 (51)	2 (2)	28 (26)	22 (21)	
**Nottingham Prognostic index**					13.267 **0.039**
Good prognostic group	30 (44)	2 (3)	22 (32)	14 (21)	
Moderate prognostic group	99 (55)	1 (1)	41 (23)	37 (21)	
Poor prognostic group	42 (58)	0 (0)	9 (13)	21 (29)	
**Ki67 expression**					8.927
Low ≤14	34 (40)	2 (2)	27 (32)	22 (26)	**0.030**
High >14	86 (59)	1 (1)	28 (19)	31 (21)	

HER2, human epidermal growth factor receptor 2 Significant P values are in **bold**

Supplementary Table 7. Multivariate analysis CDK6 expression in p53 mutated tumour

	BCSS		
	**Hazard ratio**	**95% (CI)**	***P-value***
**Parameters**			
CDK6 expression	0.51	0.27-0.98	**0.044**
Lymph node status	2.8	1.8-4.6	**<0.0001**
Tumour size	1.97	1.2-314	**0.004**

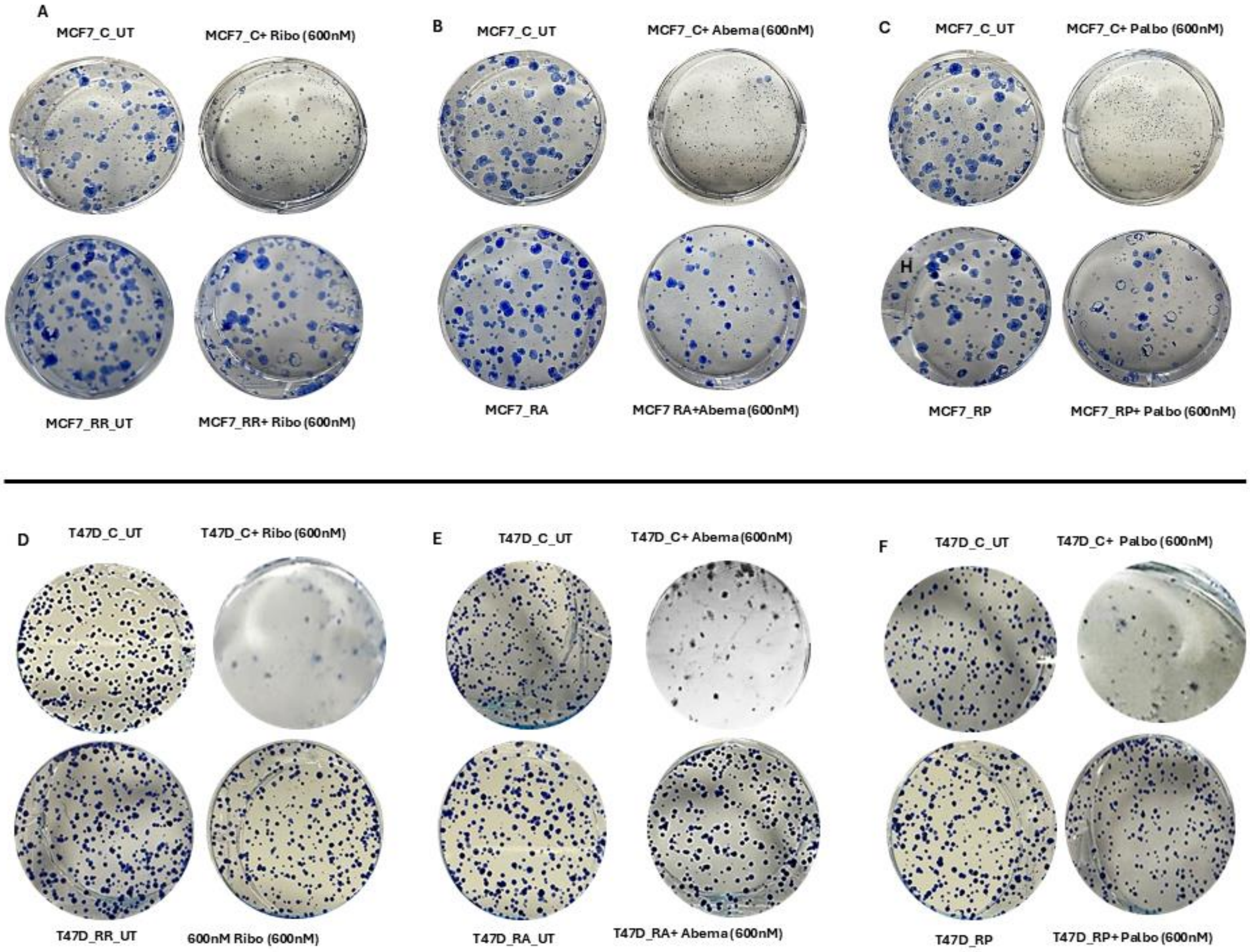

A
MCF7_C_UT
MCF7_C+ Ribo (600nM)
MCF7_RR_UT
MCF7_RR+ Ribo (600nM)
B
MCF7_C_UT
MCF7_C+ Abema (600nM)
MCF7_RA
MCF7 RA+Abema (600nM)
C
MCF7_C_UT
MCF7_C+ Palbo (600nM)
H
MCF7_RP
MCF7_RP+ Palbo (600nM)
D
T47D_C_UT
T47D_C+ Ribo (600nM)
T47D_RR_UT
600nM Ribo (600nM)
E
T47D_C_UT
T47D_C+ Abema (600nM)
T47D_RA_UT
T47D_RA+ Abema (600nM)
F
T47D_C_UT
T47D_C+ Palbo (600nM)
T47D_RP
T47D_RP+ Palbo (600nM)

Supplementary Figure 1: Clonogenics assays. (A) MCF7 control cells untreated (UT) or treated with ribociclib. (B) MCF7 control cells untreated (UT) or treated with abemaciclib. (C) MCF7 control cells untreated (UT) or treated with palbociclib. (D) T47D control cells untreated (UT) or treated with ribociclib. (E) T47D control cells untreated (UT) or treated with abemaciclib. (F) T47D control cells untreated (UT) or treated with palbociclib.

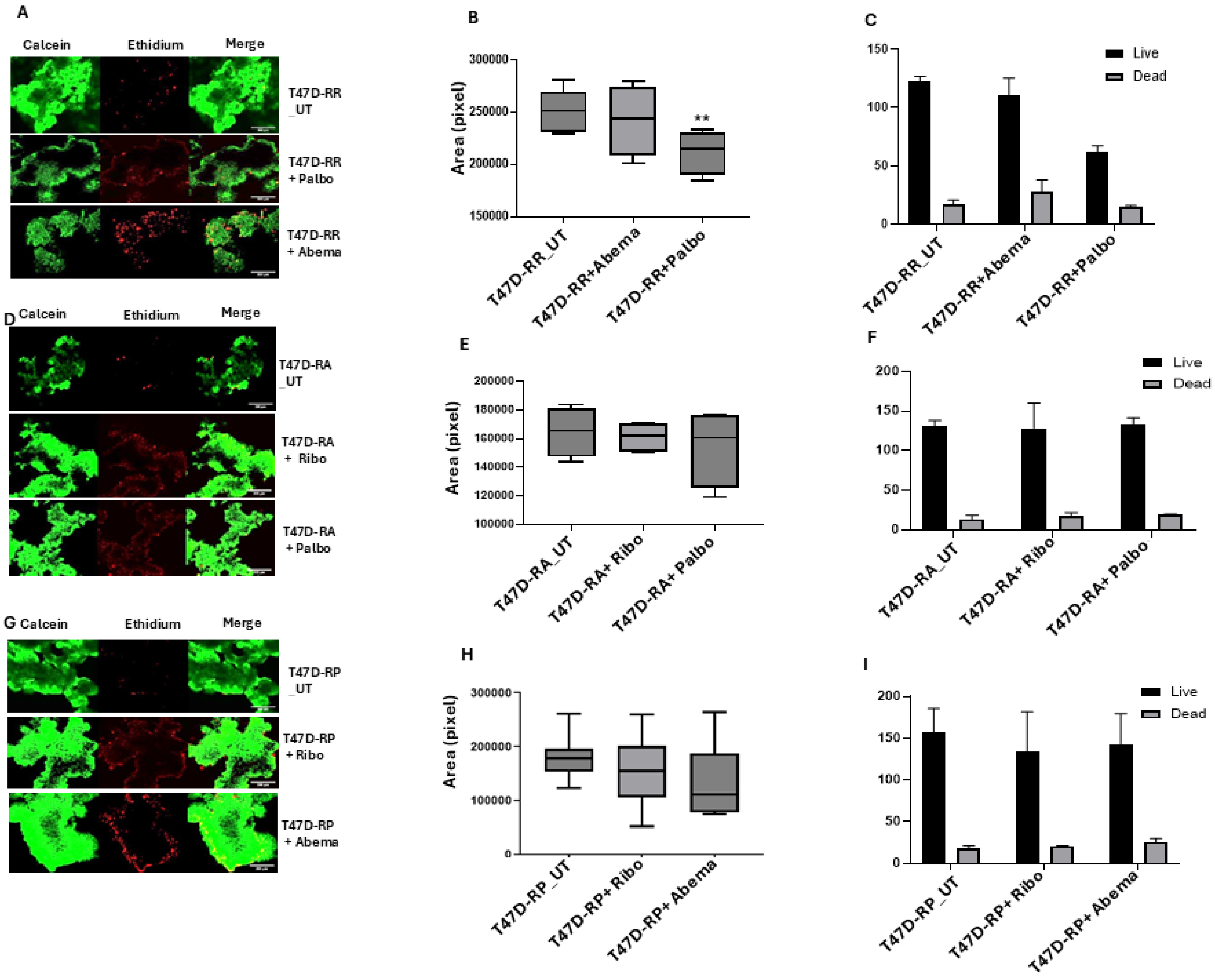
A
Calcein
Ethidium
Merge
T47D-RR _UT
T47D-RR + Palbo
T47D-RR + Abema
B
Area (pixel)
300000
250000
200000
150000
**
T47D-RR_UT
T47D-RR+Abema
T47D-RR+Palbo
C
150
100
50
0
Live
Dead
T47D-RR_UT
T47D-RR+Abema
T47D-RR+Palbo
D
Calcein
Ethidium
Merge
T47D-RA _UT
T47D-RA + Ribo
T47D-RA + Palbo
E
Area (pixel)
200000
180000
160000
140000
120000
100000
T47D-RA_UT
T47D-RA+ Ribo
T47D-RA+ Palbo
F
200
150
100
50
0
Live
Dead
T47D-RA_UT
T47D-RA+ Ribo
T47D-RA+ Palbo
G
Calcein
Ethidium
Merge
T47D-RP _UT
T47D-RP + Ribo
T47D-RP + Abema
H
Area (pixel)
300000
200000
100000
0
T47D-RP_UT
T47D-RP+ Ribo
T47D-RP+ Abema
I
200
150
100
50
0
Live
Dead
T47D-RP_UT
T47D-RP+ Ribo
T47D-RP+ Abema

Supplementary Figure 2: Cross resistance to other CDK4/6 inhibitors (A) Ribociclib resistant TD7D spheroids untreated (UT) or treated with palbociclib or abemaciclib. (B) Spheroid size in ribociclib resistant TD7D spheroids untreated (UT) or treated with palbociclib or abemaciclib. (C) % living and deal cells in ribociclib resistant TD7D spheroids untreated (UT) or treated with palbociclib or abemaciclib. (D) Abemaciclib resistant TD7D spheroids untreated (UT) or treated with ribociclib or palbociclib. (E) Spheroid size in abemaciclib resistant TD7D spheroids untreated (UT) or treated with ribociclib or palbociclib. (F) % living and deal cells in abemaciclib resistant TD7D spheroids untreated (UT) or treated with ribociclib or palbociclib. (G) Palbociclib resistant TD7D spheroids untreated (UT) or treated with ribociclib or abemaciclib. (H) Spheroid size in palbociclib resistant TD7D spheroids untreated (UT) or treated with ribociclib or abemaciclib. (I) % living and deal cells in palbociclib resistant TD7D spheroids untreated (UT) or treated with ribociclib or abemaciclib.

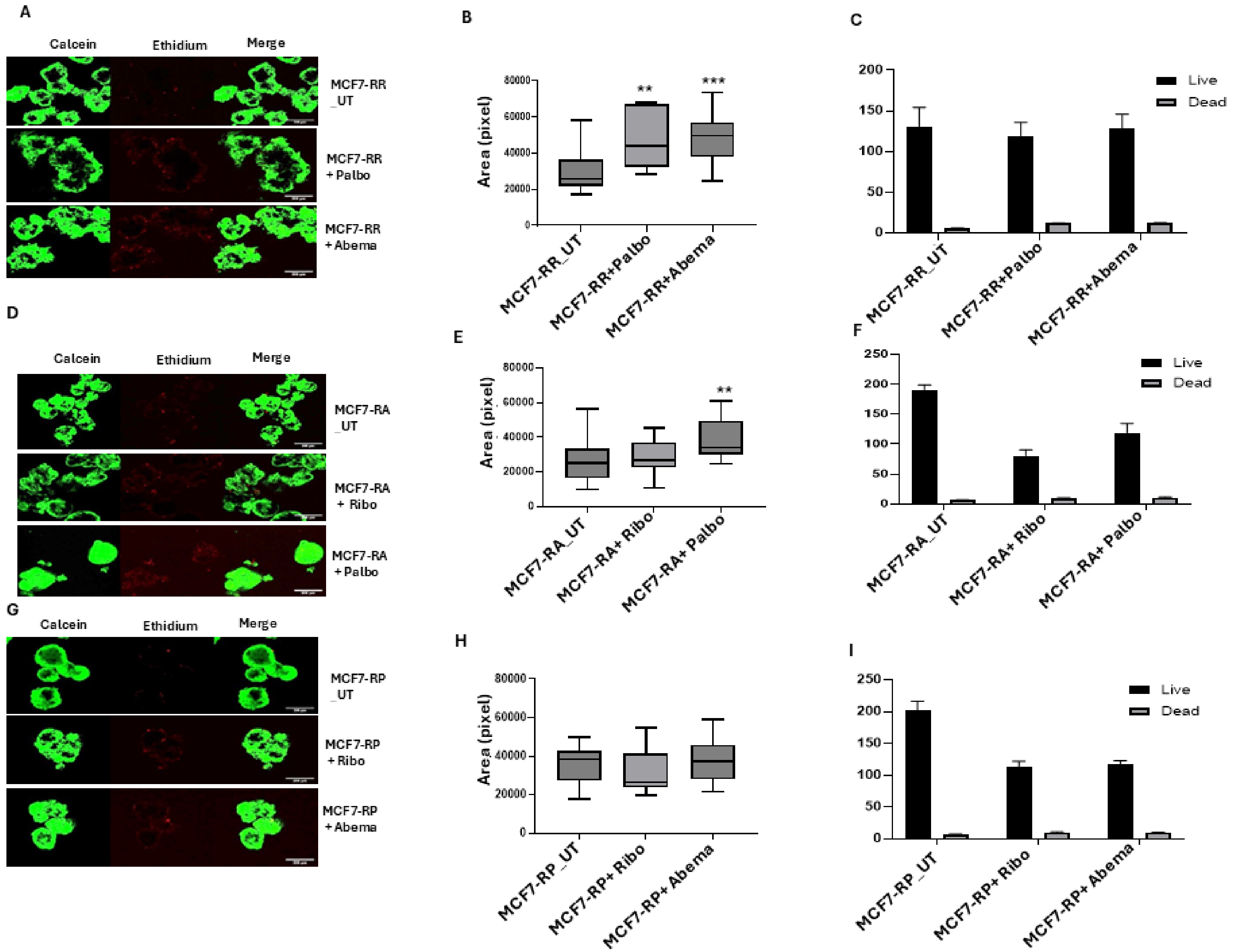

A
Calcein
Ethidium
Merge
MCF7-RR _UT
MCF7-RR + Palbo
MCF7-RR + Abema
B
Area (pixel)
80000
60000
40000
20000
0
**
***
MCF7-RR_UT
MCF7-RR+Palbo
MCF7-RR+Abema
C
200
150
100
50
0
Live
Dead
MCF7-RR_UT
MCF7-RR+Palbo
MCF7-RR+Abema
D
Calcein
Ethidium
Merge
MCF7-RA _UT
MCF7-RA + Ribo
MCF7-RA + Palbo
E
Area (pixel)
80000
60000
40000
20000
0
**
MCF7-RA_UT
MCF7-RA+ Ribo
MCF7-RA+ Palbo
F
250
200
150
100
50
0
Live
Dead
MCF7-RA_UT
MCF7-RA+ Ribo
MCF7-RA+ Palbo
G
Calcein
Ethidium
Merge
MCF7-RP _UT
MCF7-RP + Ribo
MCF7-RP + Abema
H
Area (pixel)
80000
60000
40000
20000
0
MCF7-RP_UT
MCF7-RP+ Ribo
MCF7-RP+ Abema
I
250
200
150
100
50
0
Live
Dead
MCF7-RP_UT
MCF7-RP+ Ribo
MCF7-RP+ Abema

Supplementary Figure 3: Cross resistance to other CDK4/6 inhibitors (A) Ribociclib resistant MCF7 spheroids untreated (UT) or treated with palbociclib or abemaciclib. (B) Spheroid size in ribociclib resistant MCF7 spheroids untreated (UT) or treated with palbociclib or abemaciclib. (C) % living and deal cells in ribociclib resistant MCF7 spheroids untreated (UT) or treated with palbociclib or abemaciclib. (D) Abemaciclib resistant MCF7 spheroids untreated (UT) or treated with ribociclib or palbociclib. (E) Spheroid size in abemaciclib resistant MCF7 spheroids untreated (UT) or treated with ribociclib or palbociclib. (F) % living and dead cells in abemaciclib resistant MCF7 spheroids untreated (UT) or treated with ribociclib or palbociclib. (G) Palbociclib resistant MCF7 spheroids untreated (UT) or treated with ribociclib or abemaciclib. (H) Spheroid size in palbociclib resistant MCF7 spheroids untreated (UT) or treated with ribociclib or abemaciclib. (I) % living and deal cells in palbociclib resistant MCF7 spheroids untreated (UT) or treated with ribociclib or abemaciclib.

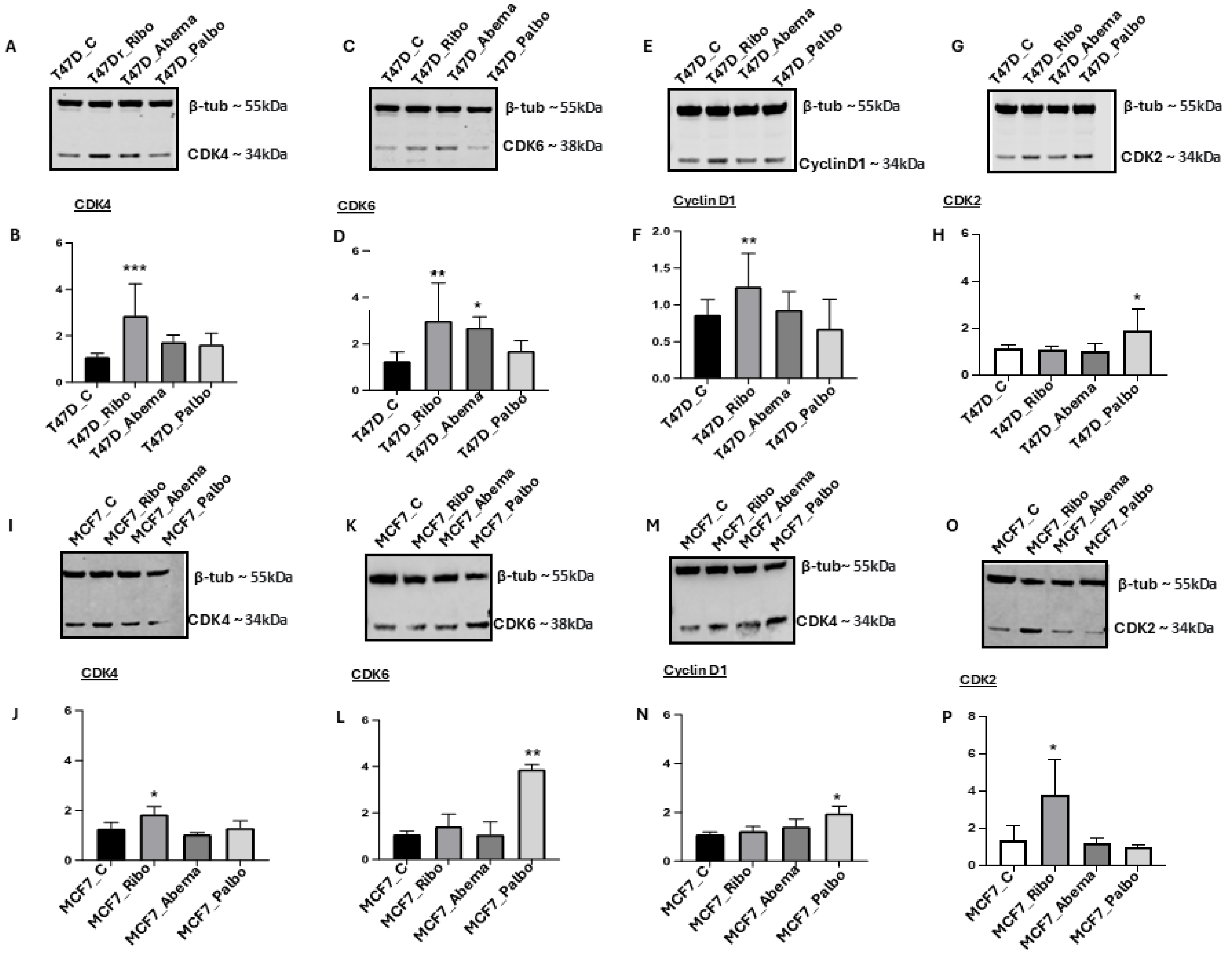

A
T47D_C
T47Dr_Ribo
T47D_Abema
T47D_Palbo
β-tub ~ 55kDa
CDK4 ~ 34kDa
C
T47D_C
T47D_Ribo
T47D_Abema
T47D_Palbo
β-tub ~ 55kDa
CDK6 ~ 38kDa
E
T47D_C
T47D_Ribo
T47D_Abema
T47D_Palbo
β-tub ~ 55kDa
CyclinD1 ~ 34kDa
G
T47D_C
T47D_Ribo
T47D_Abema
T47D_Palbo
β-tub ~ 55kDa
CDK2 ~ 34kDa
CDK4
B
***
CDK6
D
**
*
Cyclin D1
F
**
CDK2
H
*
I
MCF7_C
MCF7_Ribo
MCF7_Abema
MCF7_Palbo
β-tub ~ 55kDa
CDK4 ~ 34kDa
K
β-tub ~ 55kDa
CDK6 ~ 38kDa
M
β-tub~ 55kDa
CDK4 ~ 34kDa
O
β-tub ~ 55kDa
CDK2 ~ 34kDa
CDK4
J
*
CDK6
L
**
Cyclin D1
N
*
CDK2
P
*

Supplementary Figure 4: Western blot analysis**.** (A) CDK4 expression in T47D control and resistant cell lines. (B) CDK4 protein quantification in T47D control and resistant cell lines. (C) CDK6 expression in T47D control and resistant cell lines. (D) CDK6 protein quantification in T47D control and resistant cell lines. (E). CyclinD1 expression in T47D control and resistant cell lines (F) CyclinD1 protein quantification in T47D control and resistant cell lines. (G) CDK2 protein expression in T47D control and resistant cell lines. (H) CDK2 protein quantification in T47D control and resistant cell lines. (I) CDK4 expression in MCF7 control and resistant cell lines. (J) CDK4 protein quantification in MCF7 control and resistant cell lines. (K) CDK6 expression in MCF7 control and resistant cell lines. (L) CDK6 protein quantification in MCF7 control and resistant cell lines. (M). CyclinD1 expression in MCF7 control and resistant cell lines (N) CyclinD1 protein quantification in MCF7 control and resistant cell lines. (O) CDK2 protein expression in MCF7 control and resistant cell lines. (P) CDK2 protein quantification in MCF7 control and resistant cell lines.

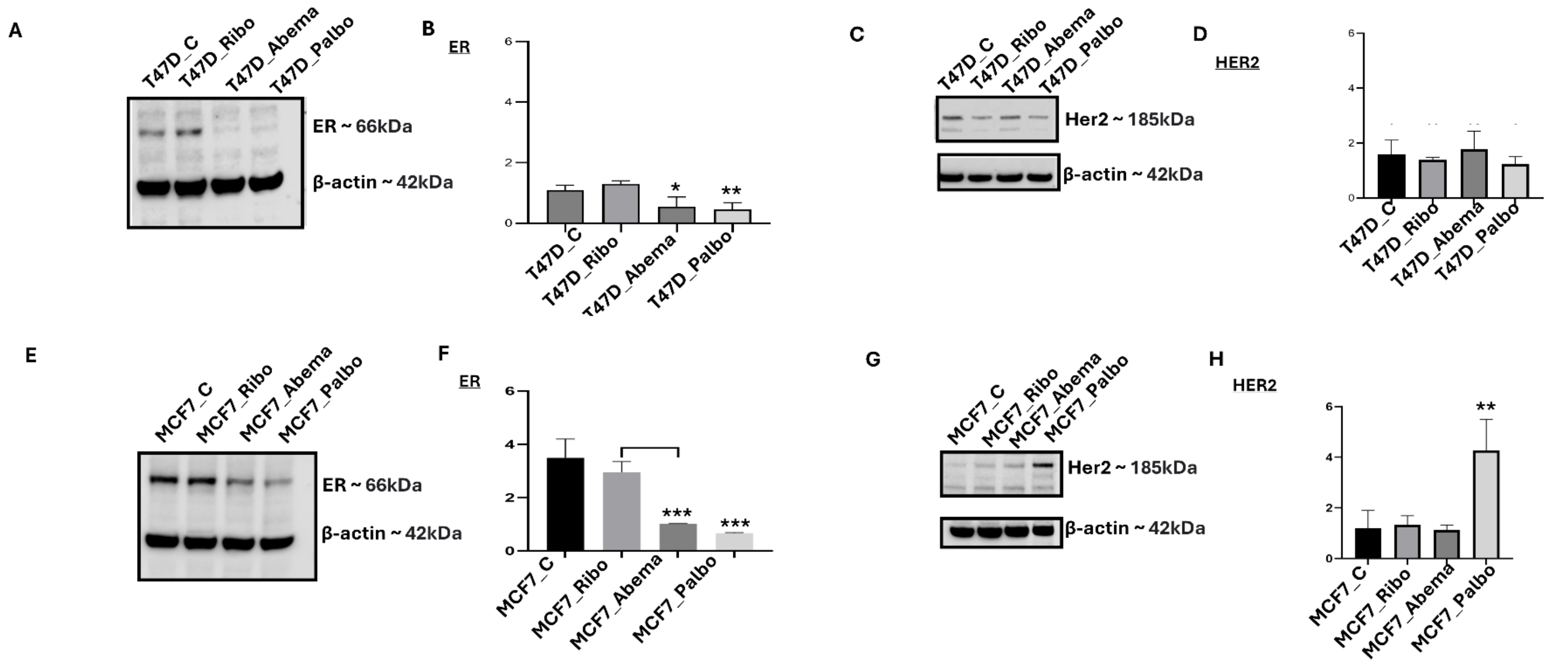

Supplementary Figure 5: Western blot analysis**.** (A) ER expression in MCF7 control and resistant cell lines. (B) ER protein quantification in MCF7 control and resistant cell lines. (C) HER2 expression in MCF7 control and resistant cell lines. (D) HER2 protein quantification in MCF7 control and resistant cell lines. (E) ER expression in T47D control and resistant cell lines. (F) ER protein quantification in T47D control and resistant cell lines. (G) HER2 expression in T47D control and resistant cell lines. (H) HER2 protein quantification in T47D control and resistant cell lines.

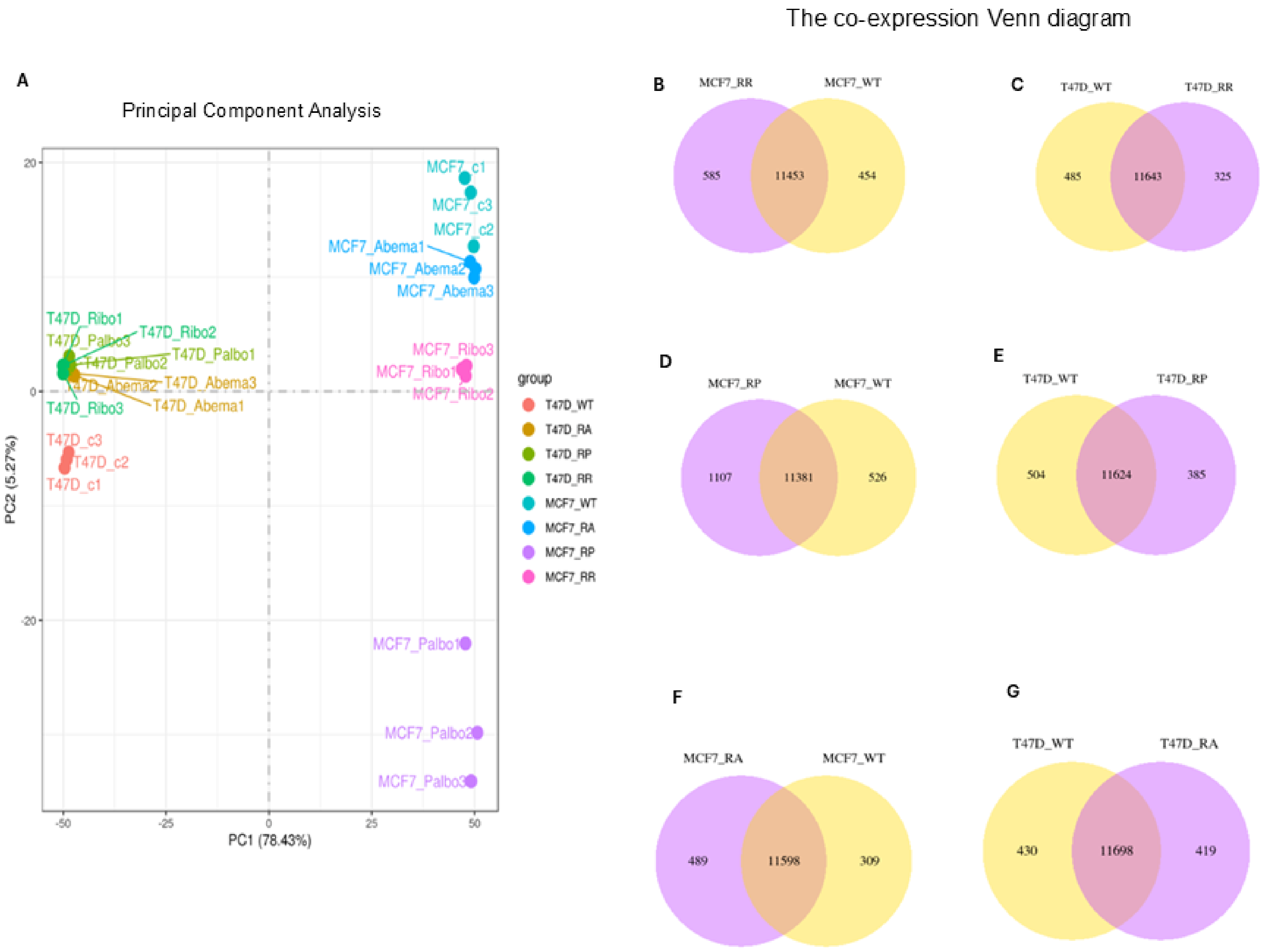
The co-expression Venn diagram
A
Principal Component Analysis
PC1 (78.43%)
PC2 (5.27%)
MCF7_c1
MCF7_c3
MCF7_c2
MCF7_Abema1
MCF7_Abema2
MCF7_Abema3
T47D_Ribo1
T47D_Ribo2
T47D_Palbo3
T47D_Palbo1
T47D_Palbo2
T47D_Abema3
T47D_Abema2
T47D_Abema1
T47D_Ribo3
T47D_c3
T47D_c2
T47D_c1
MCF7_Ribo3
MCF7_Ribo1
MCF7_Ribo2
MCF7_Palbo1
MCF7_Palbo2
MCF7_Palbo3
group
T47D_WT
T47D_RA
T47D_RP
T47D_RR
MCF7_WT
MCF7_RA
MCF7_RP
MCF7_RR
B
MCF7_RR
MCF7_WT
585
11453
454
C
T47D_WT
T47D_RR
485
11643
325
D
MCF7_RP
MCF7_WT
1107
11381
526
E
T47D_WT
T47D_RP
504
11624
385
F
MCF7_RA
MCF7_WT
489
11598
309
G
T47D_WT
T47D_RA
430
11698
419

Supplementary Figure 6: RNA sequence analysis. (A) Principal component analysis of T47D_control, resistant TD7D cells, MCF7_control, resistant MCF7 cells. (B) Venn diagram showing gene expression in MCF_RR compared to MCF7_WT (wildtype). (C) Venn diagram showing gene expression in T47D_RR compared to TD7D_WT (wildtype). (D) Venn diagram showing gene expression in MCF_RP compared to MCF7_WT (wildtype). (E) Venn diagram showing gene expression in TD7D_RP compared to T47D_WT (wildtype). (F) Venn diagram showing gene expression in MCF7_RA compared to MCF7_WT (wildtype). (G) Venn diagram showing gene expression in T47D_RA compared to T47D_WT (wildtype).

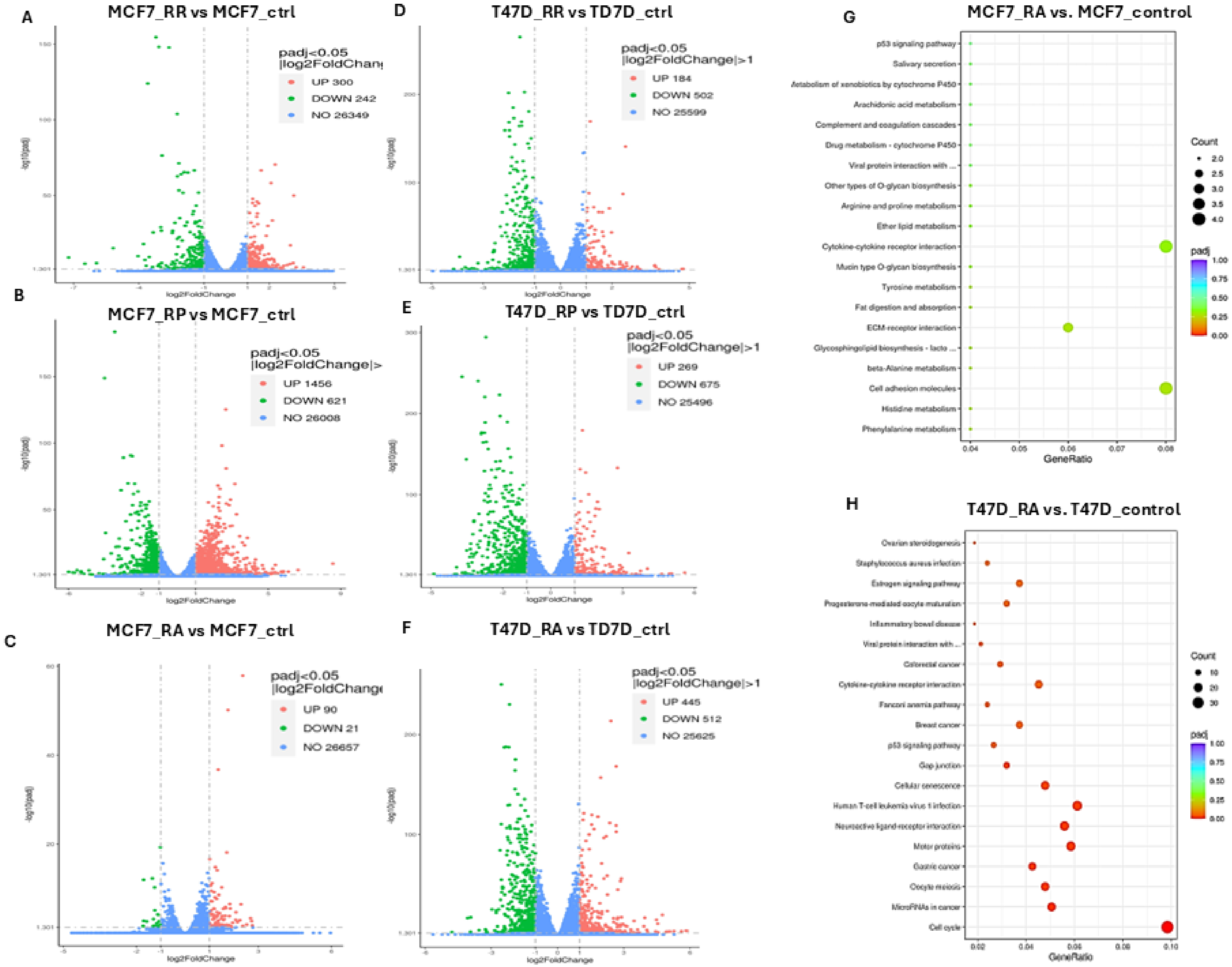
A
MCF7_RR vs MCF7_ctrl
padj<0.05
UP 300
DOWN 242
NO 26349
log2FoldChange
B
MCF7_RP vs MCF7_ctrl
UP 1456
DOWN 621
NO 26008
C
MCF7_RA vs MCF7_ctrl
UP 90
DOWN 21
NO 26657
D
T47D_RR vs TD7D_ctrl
padj<0.05
|log2FoldChange|>1
UP 184
DOWN 502
NO 25599
E
T47D_RP vs TD7D_ctrl
UP 269
DOWN 675
NO 25496
F
T47D_RA vs TD7D_ctrl
UP 445
DOWN 512
NO 25625
G
MCF7_RA vs. MCF7_control
Count
padj
GeneRatio
Cytokine-cytokine receptor interaction
ECM-receptor interaction
Cell adhesion molecules
H
T47D_RA vs. T47D_control
Count
padj
GeneRatio
Cell cycle

Supplementary Figure 7: RNA sequence analysis. (A) Volcano plot showing gene expression in MCF_RR compared to MCF7_WT (control). (B) Volcano plot showing gene expression in MCF_RP compared to MCF7_WT (control). (C(D) Volcano plot showing gene expression in T47D_RR compared to T47D_WT (control). (E) Volcano plot showing gene expression in T47D_RP compared to T47D_WT (control). (F) Volcano plot showing gene expression in T47D_RA compared to T47D_WT (control). The x-axis shows the fold change in gene expression between different samples, and the y-axis shows the statistical significance of the differences. Red dots represent up-regulation genes and green dots represent down-regulation genes. (G) Kyoto Encyclopedia of Genes and Genomes (KEGG) analysis in MCF7_RA compared to MCF7_control cells. (H) KEGG analysis in T47D_RA compared to MCF7_control cells. The most significant 20 KEGG pathways were selected for display. The abscissa is the ratio of the number of differential genes linked with the KEGG pathway to the total number of differential genes. The ordinate is KEGG Pathway. The size of a point represents the number of genes annotated to a specific KEGG pathway. The colour from red to purple represents the significant level of the enrichment.

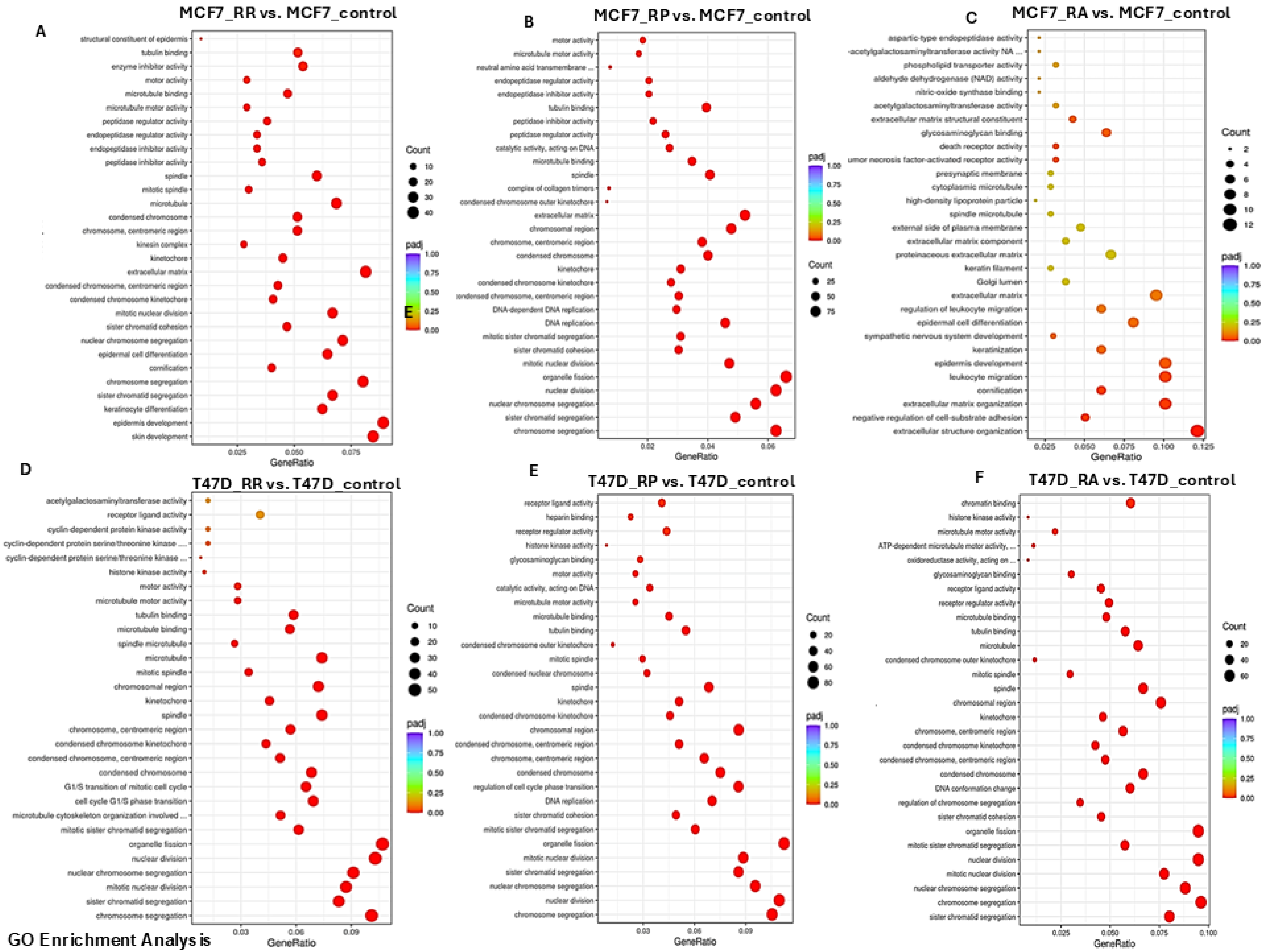

GO Enrichment Analys is

Supplementary Figure 8: Gene Ontology (GO) enrichment analysis. (A) GO analysis in MCF_RR compared to MCF7_WT (control). (B) GO analysis in MCF7_RP compared to MCF7_WT (control). (C) GO analysis in MCF7_RA compared to MCF7_WT (control). (D) GO analysis in TD74_RR compared to T47D_WT (control). (E) GO analysis in TD74_RP compared to T47D_WT (control). (F) GO analysis in TD74_RP compared to T47D_WT (control). The most significant 30 GO Terms are displayed here. The abscissa is the ratio of the number of differential genes linked with the GO Term to the total number of differential genes, and the ordinate is GO Term. The size of a point represents the number of genes annotated to a specific GO Term, and the color from red to purple represents the significant level of the enrichment.

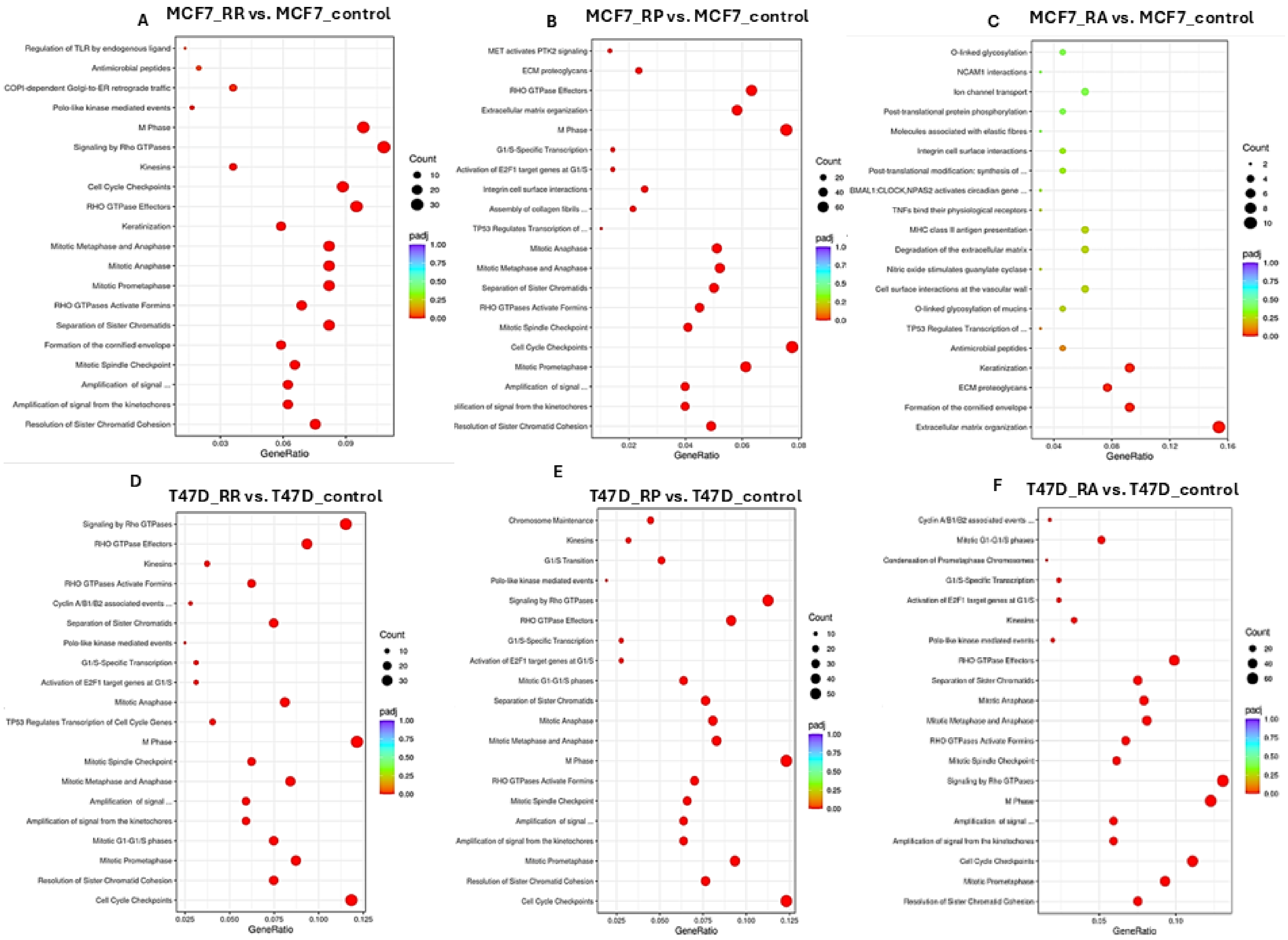

Reactome Enrichment Analysis

Supplementary Figure 9: Reactome enrichment (RE) analysis. (A) RE analysis in MCF_RR compared to MCF7_WT (control). (B) RE analysis in MCF7_RP compared to MCF7_WT (control). (C) RE analysis in MCF7_RA compared to MCF7_WT (control). (D) RE analysis in TD74_RR compared to T47D_WT (control). (E) RE analysis in TD74_RP compared to T47D_WT (control). (F) RE analysis in TD74_RP compared to T47D_WT (control). In the figure, the abscissa is the ratio of the number of differential genes linked with the Reactome pathway to the total number of differential genes, and the ordinate is Reactome Pathway. The size of a point represents the number of genes annotated to a specific Reactome pathway, and the color from red to purple represents the significant size of the enrichment.

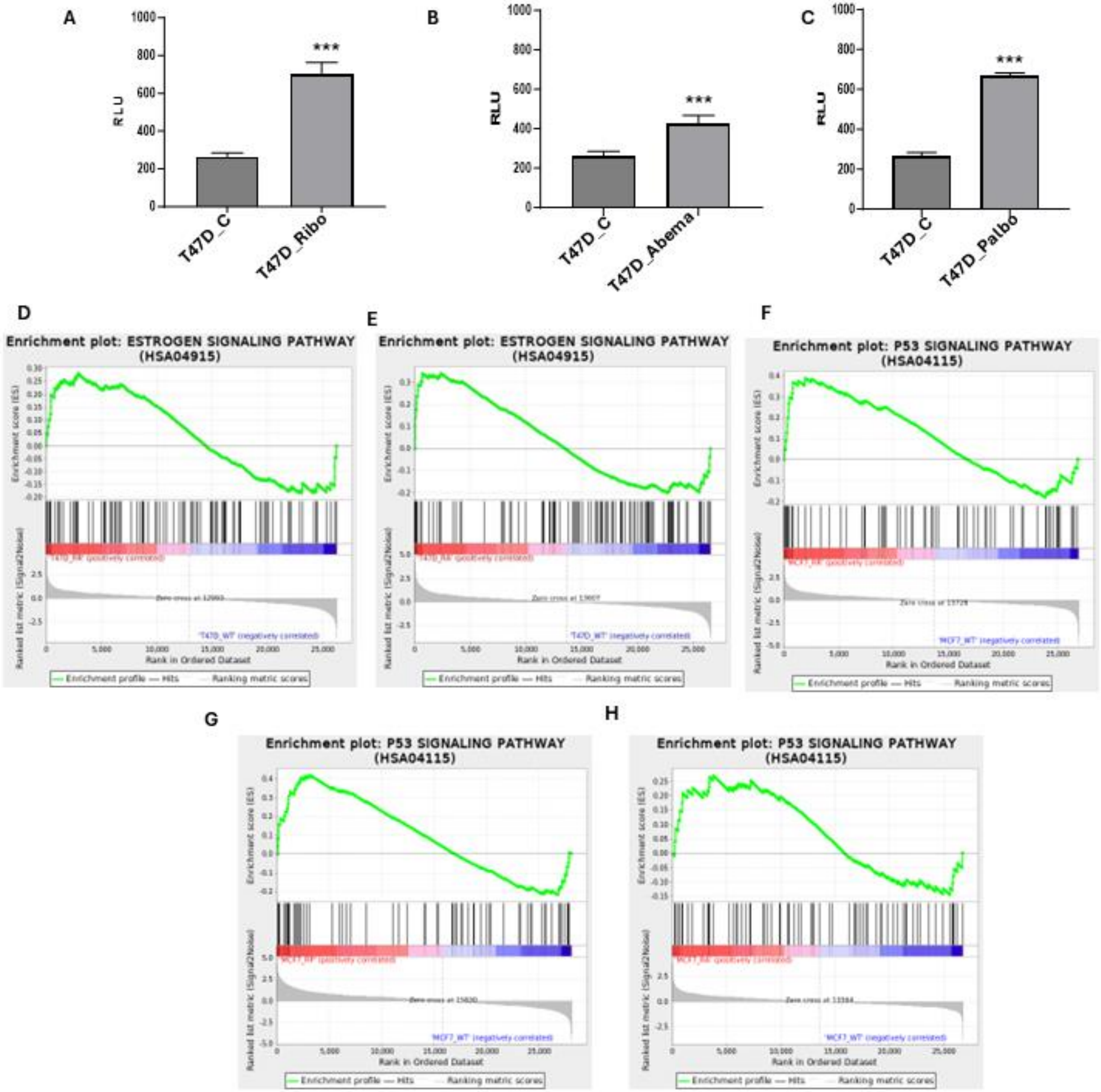
A
B
C
RLU
1000
800
600
400
200
0
***
T47D_C
T47D_Ribo
T47D_Abema
T47D_Palbo
D
E
F
G
H
Enrichment plot: ESTROGEN SIGNALING PATHWAY (HSA04915)
Enrichment plot: P53 SIGNALING PATHWAY (HSA04115)
Enrichment score (ES)
Ranked list metric (Signal2Noise)
Rank in Ordered Dataset
Enrichment profile — Hits — Ranking metric scores

Supplementary Figure 10: ER signalling and gene set enrichment analysis (GSEA) (A) ER responsive luciferase reporter assay in T47D_RR compared to T47D control. (B) ER responsive luciferase reporter assay in T47D_RA compared to T47D control. (C) ER responsive luciferase reporter assay in T47D_RP compared to T47D control. (D) Estrogen signaling GSEA in T47D_RR compared to T47D control. (E) Estrogen signaling GSEA in T47D_RA compared to T47D control. (F) p53 signaling GSEA in MCF7_RR compared to MCF7 control. (G) p53 signaling GSEA in MCF7_RP compared to MCF7 control. (H) p53 signaling GSEA in MCF7_RA compared to MCF7 control.

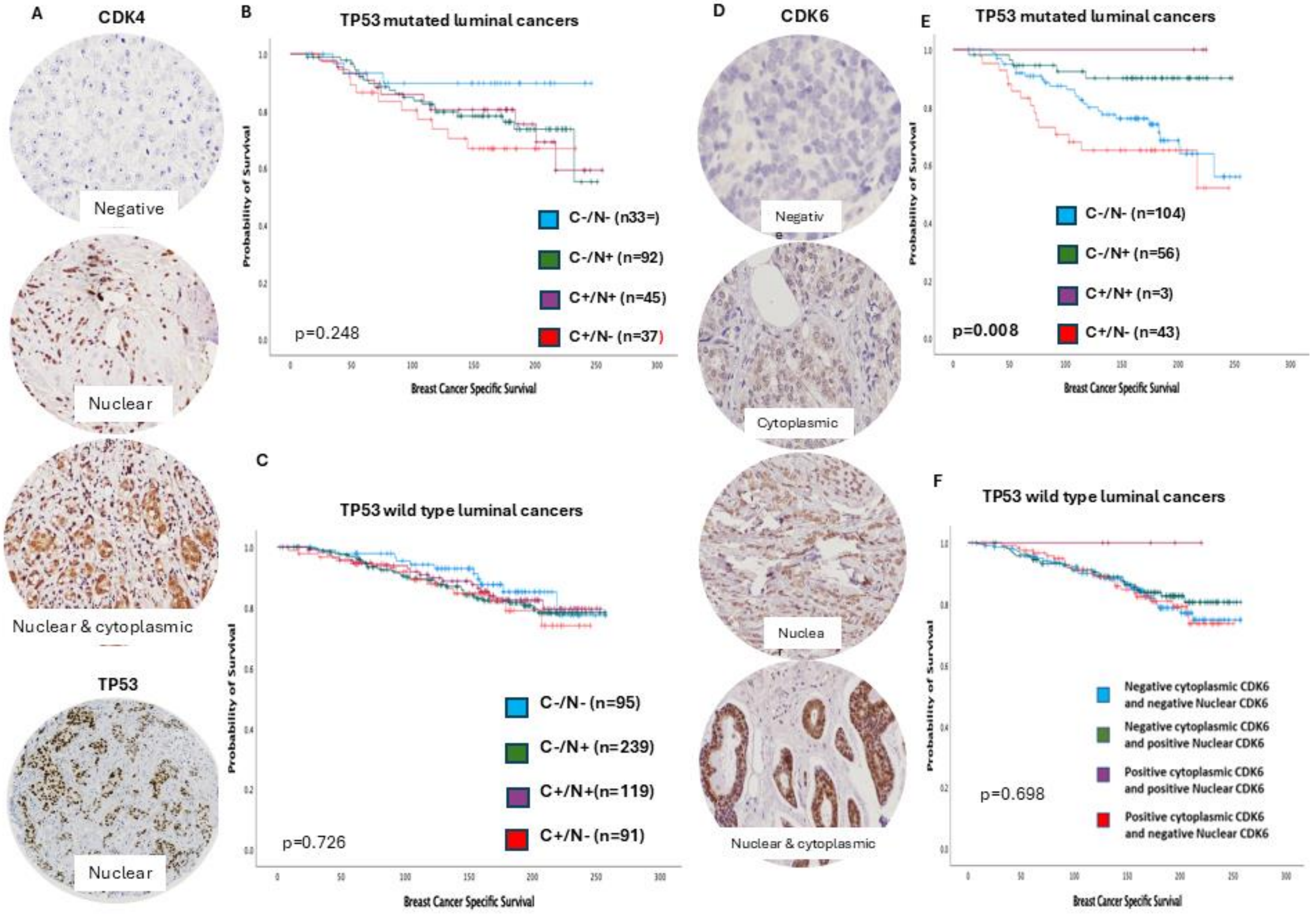
A
CDK4
Negative
Nuclear
Nuclear & cytoplasmic
TP53
Nuclear
B
TP53 mutated luminal cancers
Probability of Survival
Breast Cancer Specific Survival
C-/N- (n33=)
C-/N+ (n=92)
C+/N+ (n=45)
C+/N- (n=37)
p=0.248
C
TP53 wild type luminal cancers
Probability of Survival
Breast Cancer Specific Survival
C-/N- (n=95)
C-/N+ (n=239)
C+/N+(n=119)
C+/N- (n=91)
p=0.726
D
CDK6
Negativ e
Cytoplasmic
Nuclea r
Nuclear & cytoplasmic
E
TP53 mutated luminal cancers
Probability of Survival
Breast Cancer Specific Survival
C-/N- (n=104)
C-/N+ (n=56)
C+/N+ (n=3)
C+/N- (n=43)
p=0.008
F
TP53 wild type luminal cancers
Probability of Survival
Breast Cancer Specific Survival
Negative cytoplasmic CDK6 and negative Nuclear CDK6
Negative cytoplasmic CDK6 and positive Nuclear CDK6
Positive cytoplasmic CDK6 and positive Nuclear CDK6
Positive cytoplasmic CDK6 and negative Nuclear CDK6
p=0.698

Supplementary Figure 11: CDK4, CDK6 and p53 protein expression by immunohistochemistry (IHC) (A) Photomicrograph of CDK4 and p53 expression. (B) Kaplan–Meier curve for CDK4 nuclear/cytoplasmic co-expression and breast cancer-specific survival (BCSS) in p53 mutated luminal breast cancers. (C) Kaplan–Meier curve for CDK4 nuclear/cytoplasmic co-expression and breast cancer-specific survival (BCSS) in p53 wild-type luminal breast cancers. (D) Photomicrograph of CDK6 expression. (E) Kaplan–Meier curve for CDK6 nuclear/cytoplasmic co-expression and breast cancer-specific survival (BCSS) in p53 mutated luminal breast cancers. (F) Kaplan–Meier curve for CDK6 nuclear/cytoplasmic co-expression and breast cancer-specific survival (BCSS) in p53 wild-type luminal breast cancers.

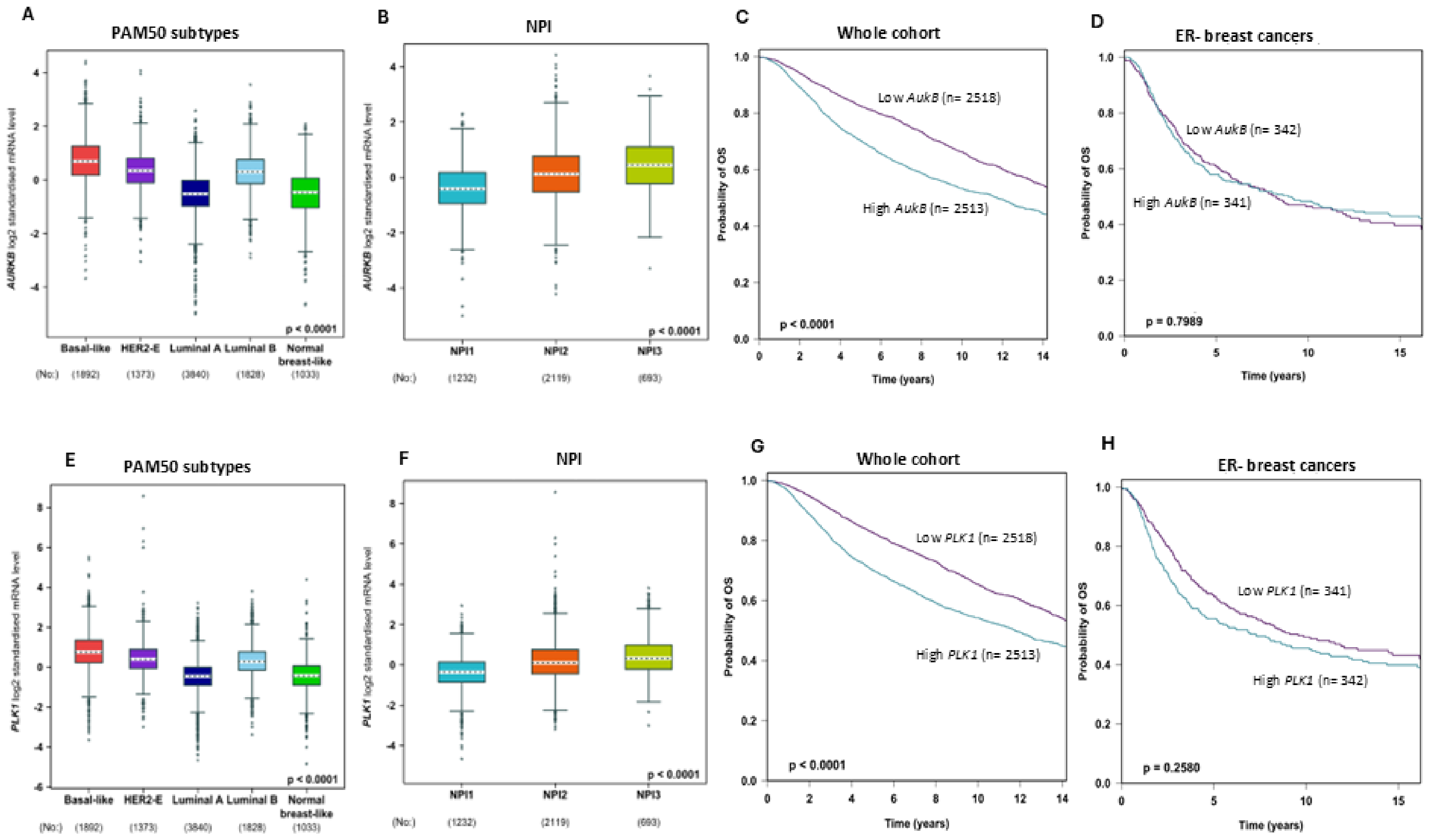

Supplementary Figure 12: *AukB* and *PLK1* transcript expression in breast cancers. (A) *AukB* transcript expression and PAM50

subtypes of breast cancer. (B) *AukB* transcript expression and Nottingham Prognostic Index (NPI) (C) Kaplan–Meier curve for *AukB* transcript expression and overall survival (OS) in the whole cohort. (D) Kaplan–Meier curve for *AukB* transcript expression and overall survival (OS) in ER- cohort. (E) *PLK1* transcript expression and PAM50 subtypes of breast cancer. (F) *PLK1* transcript expression and Nottingham Prognostic Index (NPI). (G) Kaplan–Meier curve for *PLK1* transcript expression and overall survival (OS) in the whole cohort. (H) Kaplan–Meier curve for *PLK1* transcript expression and overall survival (OS) in ER- cohort.

Full gels

Supplementary Figure 4

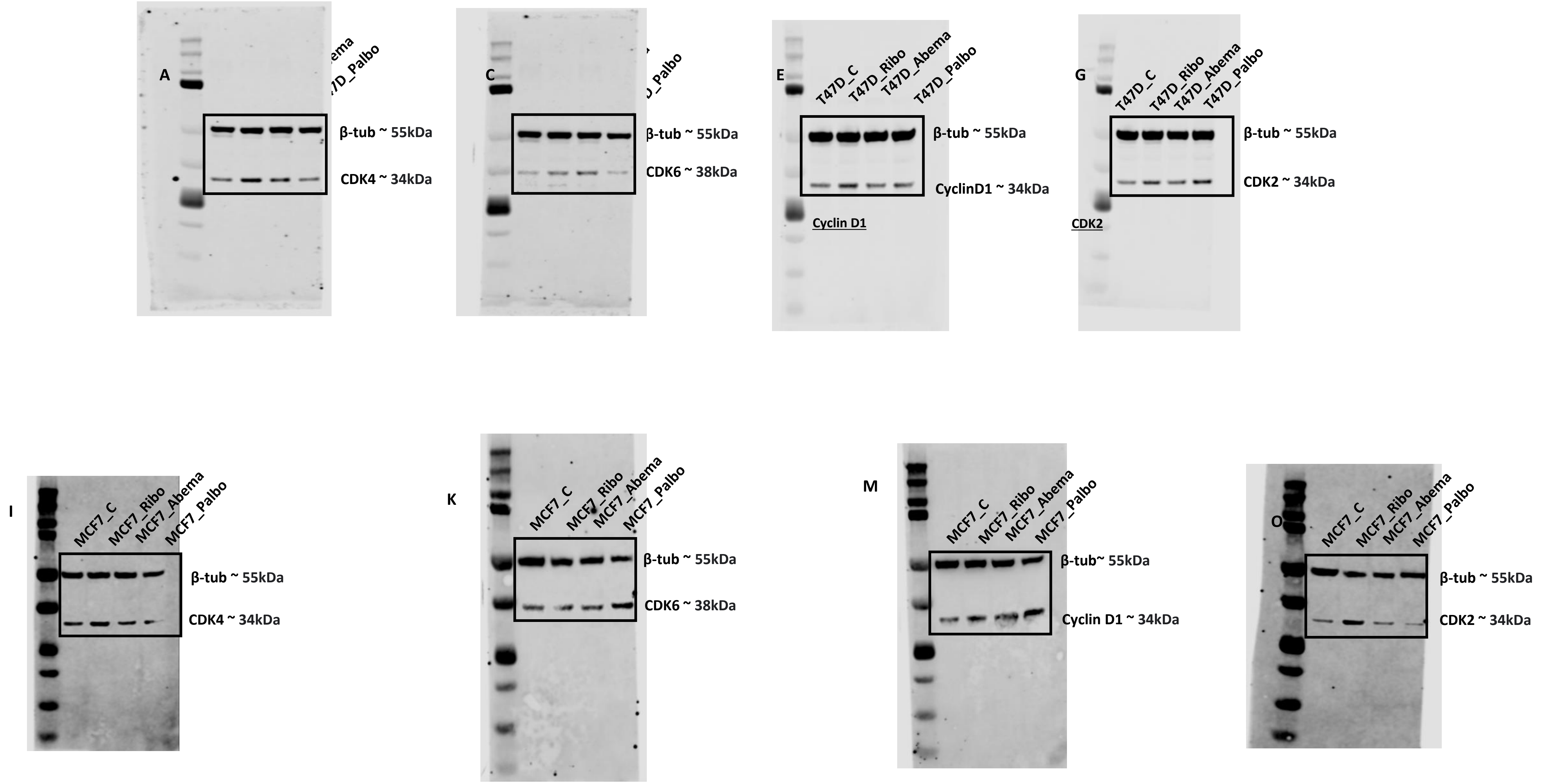

Supplementary Figure 5

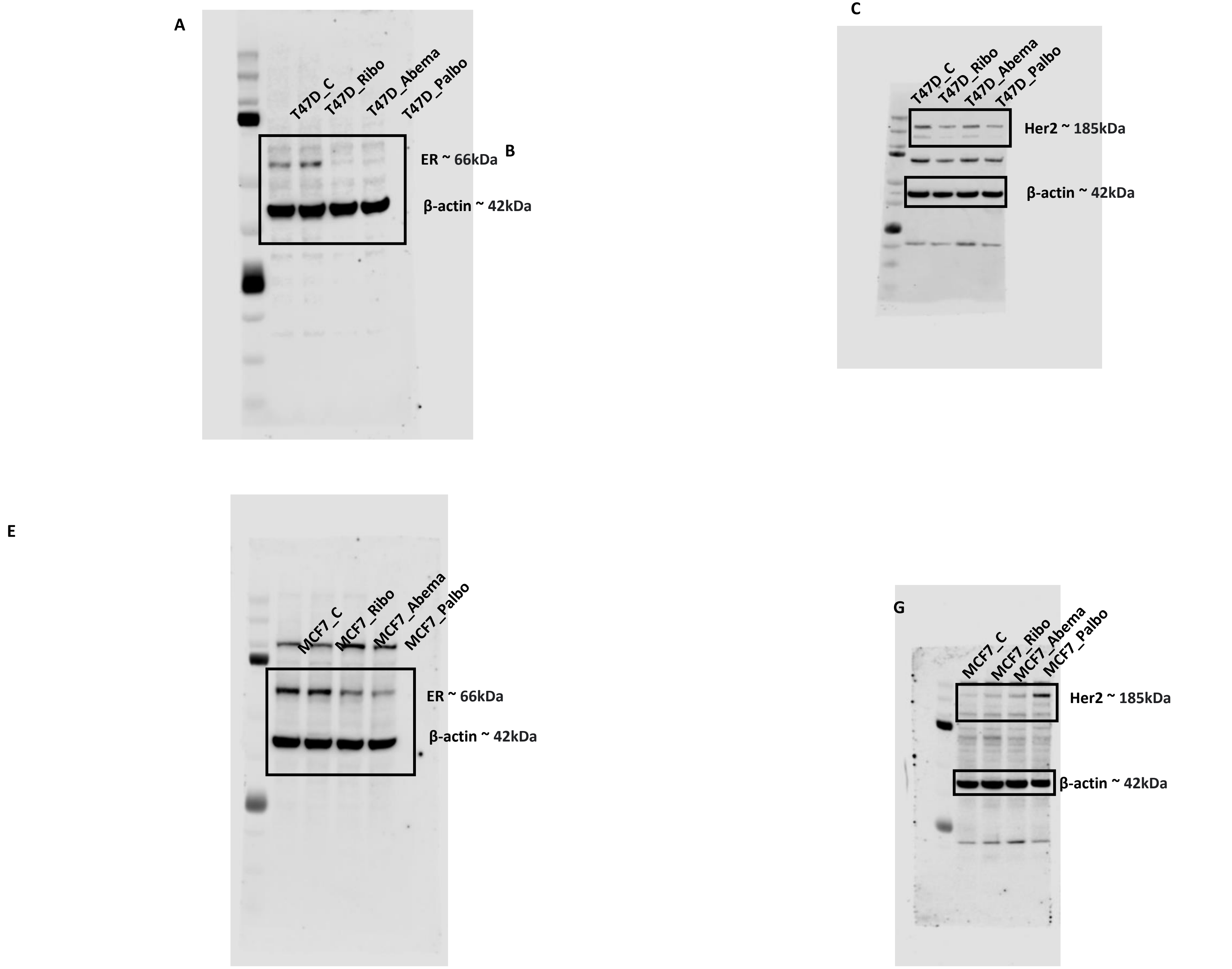

Supplement: Supplementary file 39 — Supplementary Material 39 [file 41598_2025_11052_MOESM39_ESM.pdf]
